# From curiosity to applications. A personal perspective on inorganic photochemistry

**DOI:** 10.1039/c6sc00188b

**Published:** 2016-02-12

**Authors:** Peter C. Ford

**Affiliations:** a Department of Chemistry and Biochemistry , University of California , Santa Barbara , CA 93110-9510 , USA . Email: ford@chem.ucsb.edu

## Abstract

Over the past several decades, the photochemistry and photophysics of transition metal compounds has blossomed from a relatively niche topic to a major research theme. Applications arising from the elucidation of the fundamental principles defining this field now range from probing the rates and mechanisms of small molecules with metalloproteins to light activated molecular machines. Offered here is a personal perspective of metal complex photochemistry drawn from this author's long involvement with this field. Several examples are described. Topics include characterizing key excited states and tuning these to modify chemical reactivity and/or photoluminescence properties, as well as using photoreactions as an entry to reactive intermediates relevant to homogeneous catalysts. This is followed by discussions of applying these concepts to developing precursors and precursor–antenna conjugates for the photochemical delivery of small molecule bioregulators to physiological targets.

## Abstract 2

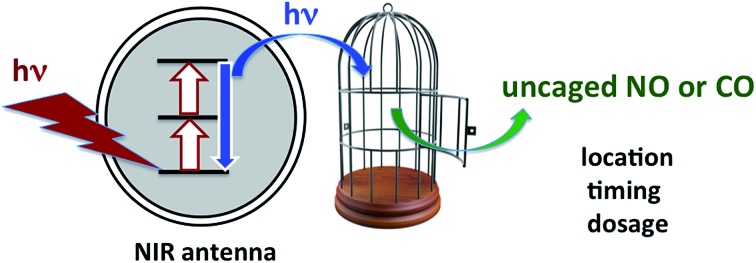
Described is an odyssey beginning with interest in colors of ruthenium(ii) complexes and evolving into photochemical uncaging of potent bioregulatory molecules.

## Introduction

**Fig. 1:**
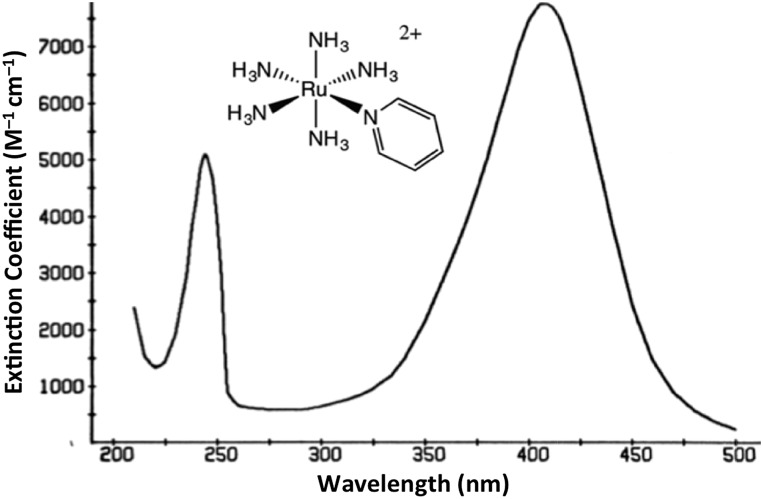
Absorption spectrum of Ru(NH_3_)_5_(py)^2+^ ion in aqueous solution.

The interaction of light and matter has long intrigued humans of all ages. Indeed this author's personal interest in the photochemistry of transition metal complexes can be traced to the qualitative observation that certain metal compounds left on the benchtop for several days looked very different than those kept in the dark for a comparable period. This was during a postdoctoral fellowship in Henry Taube's Stanford U. laboratory, where as a new convert to inorganic chemistry, I became fascinated by the bright colors of transition metal complexes. The compounds of interest were the pentaammineruthenium(ii) complexes Ru(NH_3_)_5_L^2+^ of various aromatic nitrogen heterocycles L. The absorption bands that dominate the visible absorption spectra of these Ru(NH_3_)_5_L^2+^, *e.g.*, Fig. 1, are markedly dependent on the nature of L. For example, if L is a substituted pyridine py-X, this band shifts markedly to the red when X is an electron accepting substituent and to the blue when X is an electron donor.^1^ This and related observations drove me to study C. K. Jørgensen's book on metal complex spectroscopy^2^ and to learn sufficient group and ligand field theory to interpret these absorption bands as metal-to-ligand charge transfer (MLCT) transitions.^1^

It was with these materials that I first noted light induced color changes and learned to store my samples in the dark. Furthermore, curiosity about the underlying causes led me to begin investigating the photoreactions of the Ru(ii) ammine complexes, once I joined the chemistry faculty of the University of California, Santa Barbara (UCSB). I was fortunate that several UCSB colleagues were studying gas phase photoreactions and, moreover, were willing to teach this novice some nuances of photochemical science as it was then practiced. To set the context, I should emphasize that there had already been significant activity investigating photoreactions of metal complexes. Much of this work has been summarized in books by Balzani and Carassiti (1970)^3^ and by Adamson and Fleischauer (1974),^4^ both of which remain valuable resources. In addition, it would be remise not to draw special attention to the iron(iii) oxalate-based actinometry developed by Hatchard and Parker in the UK (1956).^5^ This provided a convenient and sensitive method for measuring the intensities of the light at the location where solution phase photochemical reactions occur. It is a tool that continues to be in use many decades later.

Transition metal photochemistry continues to evolve, especially as new tools or potential new applications are realized. Offered here is a personal and not very comprehensive perspective of this evolution organized around the author's focus on the reactions and mechanisms that are triggered by light.

## Fundamental mechanistic photochemistry

### Excited states

The first question one might ask when considering the solution photochemistry of a complex ion like Ru(NH_3_)_5_(py-X)^2+^ is “what excited states (ES) are involved?” From the orbital parentage of these ES, one might develop a qualitative view of expected reaction trajectories. Given the power of density functional theory (DFT) computations now accessible, such visualization might seem primitive, but it does provide intuition of the type that has made the practice of chemistry a very human activity. Stepping back to our earliest photochemical investigations, neither DFT nor laser flash photolysis were readily available. As a consequence, mechanistic conclusions were largely built on determining photoreaction products and quantum yields and on comparing these data to the electronic spectroscopic properties.

**Fig. 2:**
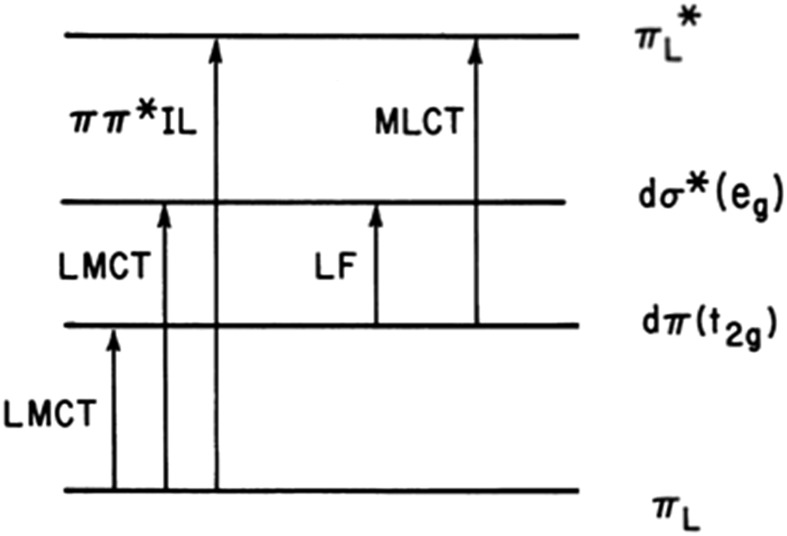
Representation of one-electron transitions between the MOs of a mononuclear metal complex ML_6_ in *O*_h_ symmetry. LF: ligand field transition; LMCT: ligand to metal charge transfer; MLCT: metal to ligand charge transfer; IL: intraligand or ligand centered.

Fig. 2 illustrates the types of one-electron transitions that are commonly used to assign absorption bands and excited states in mononuclear metal complexes. Those most characteristic of transition metal complexes with partially filled d-orbitals are absorption bands representing excitation between “d” orbitals that are split by mixing with σ-donor and π-donor and acceptor orbitals of the ligands. These “d–d” ligand field (LF) transitions involve molecular orbitals (MOs) that are largely metal in character, hence the resulting ES are often referred to as metal-centered excited states (MC*). In a centrosymmetric complex, LF bands are Laporte forbidden and typically have relatively low extinction coefficients. In contrast, metal-to-ligand charge transfer (MLCT) and ligand-to-metal charge transfer (LMCT) bands are much more intense owing to the dipole moment change between the ground and excited states. The fourth type denoted in this figure is a ligand centered or intraligand (IL) transition, represented here as π_L_ → π*L excitation. While these one-electron designations provide a convenient framework for addressing the natures of the relevant ES, theoretical calculations have long shown that there is much more mixing of the excited state characters than implied by Fig. 2.

For Ru(NH_3_)_5_(py-X)^2+^ and related complexes such as Ru(NH_3_)_5_(pz)^2+^ (pz = pyrazine), the strong absorption bands (*ε* ∼ 10^4^ M^–1^ cm^–1^) seen in the visible spectra were assigned as spin-allowed (singlet to singlet) MLCT transitions, while those in the ultraviolet region (UV) as IL (π_L_ → π*L) in character. Where would one expect to find the LF absorptions for these low spin d^6^ complexes? As an empirical model, one might consider the spectrum of Ru(NH_3_)_6_^2+^, which does not include any π-unsaturated ligands. The electronic spectrum of this octahedral complex displays two relatively weak absorption bands at 390 nm (*ε* = 35 M^–1^ cm^–1^) and 275 nm (640 M^–1^ cm^–1^) as predicted by group theory (^1^A_1g_ → ^1^T_1g_ and ^1^A_1g_ → ^1^T_2g_)^7^ (note: the higher intensity of the second band has been attributed to mixing with a charge transfer to solvent transition, CTTS).^7^ Analogous LF bands might be expected at comparable energies for the Ru(NH_3_)_5_(py-X)^2+^ ions, but they are obviously obscured by the much stronger MLCT bands.

### Quantum yields and ES dynamics

The quantum yield is basically an efficiency measurement and can be defined for a specific photoprocess *i* as shown in eqn (1). Note that *Φ*_*i*_ is unit-less. An efficient process has a quantum yield near 1 (or higher if a chain reaction results); however, as discussed below, there are many reasons why *Φ*_A_ might be much smaller. A key point here is the nature of the denominator, since photoreactions will not occur unless light is absorbed. Quantum yields are typically measured at a specific wavelength of irradiation (*λ*_irr_), so this concept is less precise when a broadband excitation source such as sunlight is used.1
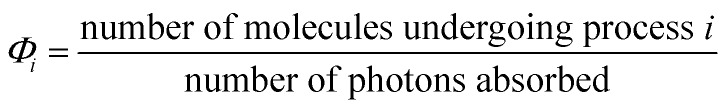



With certain applications, for example, the uncaging of a drug at a physiological target, the overall *rate* of the photochemical reaction is of key interest. The photoreaction rate equals *Φ*_*i*_ × *I*_a_, where *I*_a_ is the intensity of the light *absorbed*, typically in units of einsteins per unit time (1 einstein = 1 mole of photons) or in einsteins per unit volume per unit time. For a solution where the photoreactant is the only species absorbing the incident light, *I*_a_ = *I*_0_(1 – 10^–Abs(*λ*)^), where Abs(*λ*) is the solution absorbance at *λ*_irr_ and equals the product of the molar concentration of the photoactive species (*c*), the molar extinction coefficient (*ε*_*λ*_, in L mol^–1^ cm^–1^) at that *λ* and the path-length of the cell (in cm). *There is no unimolecular rate constant associated with this process*.

Taking for example Ru(NH_3_)_5_(py)^2+^, continuous photolysis of this ion with 405 nm light in ambient temperature, aqueous solution resulted in the photosubstitution reactions depicted in eqn (2).^8^ The respective quantum yields for pyridine and ammonia substitution *Φ*_py_ and *Φ*_NH_3__ were measured as 0.045 and 0.063 for *λ*_irr_ = 405 nm. This excitation wavelength is close to the *λ*_max_ of the spin-allowed MLCT band (407 nm), so one can be assured that the vast majority of the photons absorbed lead to MLCT excitation.2
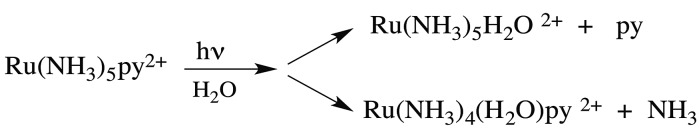



Are the observed photosubstitution reactions consistent with that expected for an MLCT excited state, which can simplistically be represented as having an oxidized metal center and a reduced pyridine, [Ru^III^(py^–^)]^2+^? Given that low spin d^5^ Ru(iii) complexes are not very thermally labile, this behavior is not what one might expect for the MLCT* state. In contrast, the MC* states, which involve promoting an electron from a non-bonding or even π-bonding orbital to one which is σ* relative to the metal–ligand bonds would appear to be logical precursors of ligand photosubstitutions. Consistent with this argument, the hexaammine complex Ru(NH_3_)_6_^2+^ is comparably labile toward photodissociation of NH_3_ (*Φ*_NH_3__ = 0.27 for *λ*_irr_ = 405 nm).^7^ Drawing such a conclusion does, however, presume that the mechanism for ES ligand labilization is predominantly dissociative in character. In support of this view, a recent TDDFT calculation on the metal centered ^3^MC* state of Ru(NH_3_)_5_(py)^2+^ confirms that this state is nearly dissociative in character.^9^

**Fig. 3:**
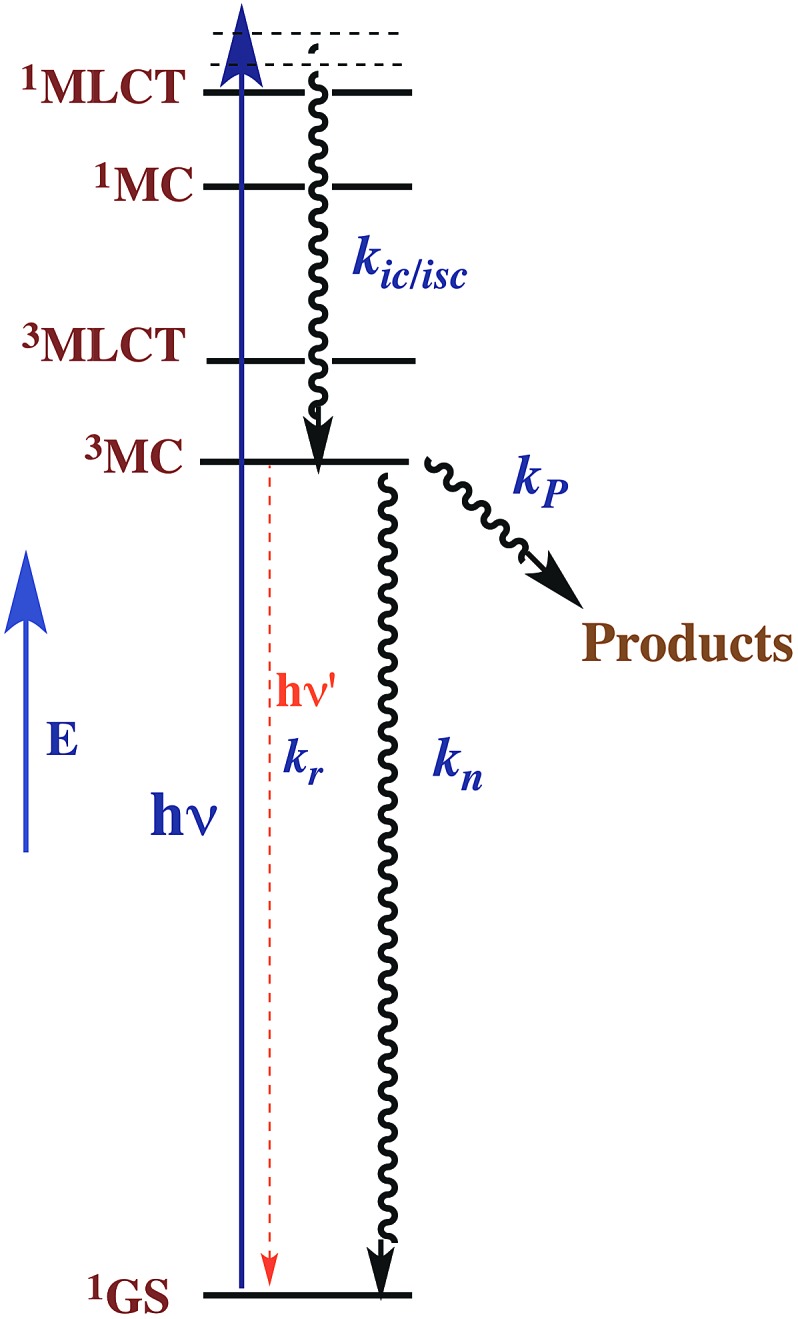
Proposed ES diagram to explain photoaquation of aq. Ru(NH_3_)_5_(py)^2+^ upon irradiation of MLCT bands. *k*_P_ is the rate constant for reactions to photoproducts. *k*_ic_/*i*_sc_, *k*_n_ and *k*_r_ are rate constants for internal conversion/intersystem crossing to a lowest energy metal centered excited state, nonradiative deactivation to the ground state ^1^GS and radiative deactivation (emission), respectively.

This reasoning led to our proposal that the ES responsible for the reactions noted in eqn (2) was the result of rapid internal conversion/intersystem crossing from MLCT states populated by excitation to spectrally unobserved MC* states as illustrated in Fig. 3.^10^ The observation that photolabilization of aq. Ru(NH_3_)_5_(py)^2+^ is largely independent of the excitation wavelength suggested that a common reactive excited state, presumably the lowest energy excited state(s) (LEES), is responsible for these photoreactions. Another consequence of this model is the prediction that we can use ligand substituents to tune the excited state energies to where the ^3^MLCT is the LEES. In that case, there should be marked decreases in the observed photolability.

Our ES “tuning model” was thus demonstrated by examining the photoreactions of a series of Ru(NH_3_)_5_(py-X)^2+^ complexes.^11^ Electron-withdrawing substituents that shift the MLCT band to *λ*_max_ values > 470 nm led to dramatically less photolability than those with *λ*_max_ values at shorter wavelengths. Similar MLCT/MC state tuning approaches have since been used with a number of systems including those based on the Ru(ii) polypyridyl complexes of interest with regard to solar energy conversion devices^12^ and similar Ir(iii) phenylpyridyl complexes of interest for organic light emitting diodes (OLED) applications.^13^

The observation that photoreaction quantum yields *Φ*_P_ for aq. Ru(NH_3_)_6_^2+^ and Ru(NH_3_)_5_(py)^2+^ are considerably less than unity is explained by the Jablonski diagram shown in Fig. 3. Radiative and nonradiative pathways for ES deactivation to the ground state (GS) compete with the chemical pathways leading to products. In this simplified diagram, all three pathways are represented as occurring from the lowest energy ES of metal complex. However, one should keep in mind that with organic molecules and other compounds of the lighter elements, intersystem crossing (ISC) rates are often not competitive with direct radiative (fluorescence) and non-radiative deactivation to the GS. Furthermore, Franck–Condon states formed directly by excitation from the GS may undergo processes, including ISC, faster even than vibrational relaxation.^14^ Thus, the ES kinetics can be quite complex owing to the numerous states and deactivation pathways potentially involved (note: for compounds of lighter elements, *e.g.*, organic dyes, internal conversion to the lowest energy ES of a particular spin state is typically much faster than the forbidden ISC to an ES of a different spin state. However, the much greater spin orbit coupling with complexes of heavier elements tends to break down this pattern).

For illustrative purposes, we will continue discussion of quantum yields based on the simple model described by Fig. 3, where a single state (or collection of ES) is populated by rapid and highly efficient internal conversion and ISC from states initially formed by excitation. For such a system, where decay occurs only by first order ES processes, *Φ*_*i*_ can also be described (eqn (3)), where *k*_P_ is the rate constant for photoproduct formation, while *k*_n_ and *k*_r_ represent those for nonradiative deactivation to the ground state and radiative deactivation (emission), respectively. In the case of aq. Ru(NH_3_)_5_(py)^2+^ emission is at best very weak, and the photoproduct quantum yield *Φ*_P_ is less than 0.5, so the dominant LEES pathway must be nonradiative deactivation.

3 *Φ*_*i*_ = *k*_*i*_/(*k*_P_ + *k*_n_ + *k*_r_)

Excited state lifetimes can be measured either by transient absorption, transient bleaching or luminescence methods. If the decay pathways from the LEES are unimolecular as implied by Fig. 3, then the observed lifetime *τ* equals (∑*k*_*i*_)^–1^, where in this case *τ* = (*k*_P_ + *k*_n_ + *k*_r_)^–1^. Thus eqn (3) can be rewritten eqn (4), and excited state rate constants for individual pathways can be calculated from *k*_*i*_ = *Φ*_*i*_*τ*^–1^. However, it must be emphasized that, while this approach often applies to complexes of the heavier transition metals, the kinetics are more complicated when more than one ES is responsible for the chemical or photoluminescence behavior.

4 *Φ*_*i*_ = *k*_*i*_*τ*

It was long thought that the Ru(NH_3_)_5_(py-X)^2+^ complexes did not display any emission either in solution or as solid salts. This conclusion is a matter of detection sensitivity given the einstein principle that every excited state undergoes spontaneous emission.^15^ The inherent intensities of such luminescence are determined by the competing processes that deplete that ES. Ultrafast flash photolysis studies by Winkler *et al.*^16^ and others^17^ did not detect emission, but with transient bleaching measurements did demonstrate ES lifetimes from <20 ps to ∼200 ps in ambient temperature solution. The longer values of *τ* were seen for those complexes for which the LEES are MLCT in character. Recently, Endicott and coworkers^9^ detected very weak emissions (*Φ*_r_ ∼ 5 × 10^–5^) from several of these complexes in 77 K organic glasses. Under these conditions, lifetimes were found to be on the 0.1–3 μs timescale, so the weak emissions were attributed to very low values of *k*_r_ rather than to short lifetimes under these conditions.

**Fig. 4:**
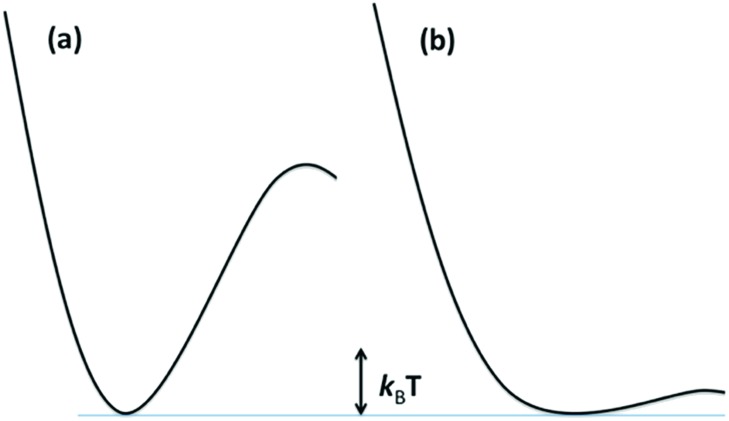
Potential surfaces for (a) a bound excited state where the barrier is >*k*_B_*T* and (b) a dissociative ES where the barrier is <*k*_B_*T*. Since *k*_B_*T* is temperature dependent, if any barrier is present, an ES that is dissociative at a high *T*, may behave as a bound state at low *T*.

The reaction dynamics implied by Fig. 3 and eqn (3) assumes that the lowest energy ES lies on potential surfaces with clearly defined minima. Such states are “bound” ES, for which there is an energy barrier larger than *k*_B_*T*, (*k*_B_ being the Boltzmann constant) to decay along any relevant reactive coordinate (Fig. 4). It is also possible to have a “dissociative” state, where the barrier is less than *k*_B_*T*. The latter would have exceedingly short lifetimes, and it is not correct to treat their dynamics by classical kinetics methods.

## Coordination compounds

There have been a number of quantitative photochemical studies of coordination compounds, although the earlier studies primarily focused on those that were thermally stable in solution. Consequently, d^3^ (primarily Cr(iii) complexes), low spin d^6^ (Fe(ii), Ru(ii) Os(ii), Co(iii), Rh(iii), Ir(iii) and some Pt(iv) complexes) and low spin d^8^ complexes (*e.g.*, Rh(i), Ir(i) and Pt(ii)) received the most attention, although this list is not comprehensive. Described here are several Rh(iii) ammine complexes for which the detailed ligand photosubstitution dynamics and mechanisms were studied in this laboratory. These provide a valuable case study on the reactions of the metal-centered excited states of low spin d^6^ complexes, an electronic configuration of metal systems often found in applications such as dye sensitized solar cells (DSSCs) and OLEDs.

### Rhodium(iii) amine complexes

**Fig. 5:**
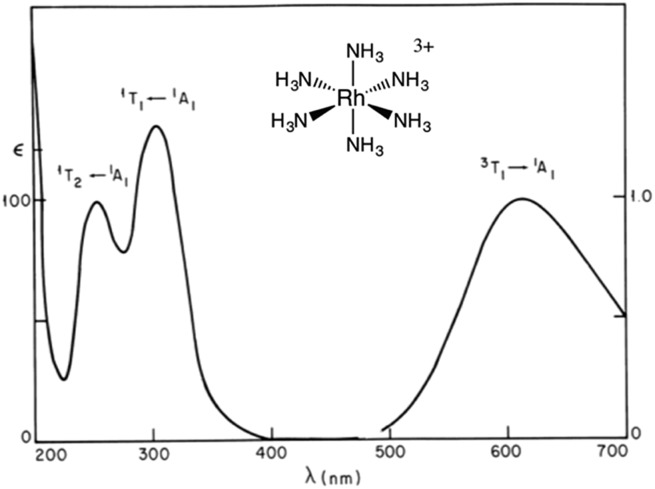
Absorption (left) and emission (right) spectra of Rh(NH_3_)_6_^3+^ in aqueous solution.

The importance of MC* states in defining the photolability of the Ru(ii) species even when initial excitation is MLCT led us to focus attention on the analogous photochemistry of the isoelectronic Rh(iii) ammine complexes such as the Rh(NH_3_)_6_^3+^ and Rh(NH_3_)_5_Cl^2+^ ions.^18^ The absorption spectra of these rhodium ammine complexes are dominated by the spin-allowed LF bands (^1^A_1_ → ^1^T_2_, ^1^A_1_ → ^1^T_1_ for the *O*_h_ complex Rh(NH_3_)_6_^3+^, Fig. 5) predicted by group theory, while the emission spectra are characteristic of that expected from the lowest energy ^3^MC* states (^3^T_1_ → ^1^A_1_ for Rh(NH_3_)_6_^3+^). Our entry into these studies also corresponded to our collaborations with R. J. Watts and D. S. Magde, with whom we were able to conduct ns and ps pulsed laser experiments that allowed determining for the first time the emission and transient absorption lifetimes of the lowest energy MC* states in fluid solutions.^19^–^22^ In this manner we were also able to probe the ^3^MC* state ligand substitution and nonradiative decay mechanisms with hydrostatic pressure effects in collaboration with R. van Eldik.^23^

**Fig. 6:**
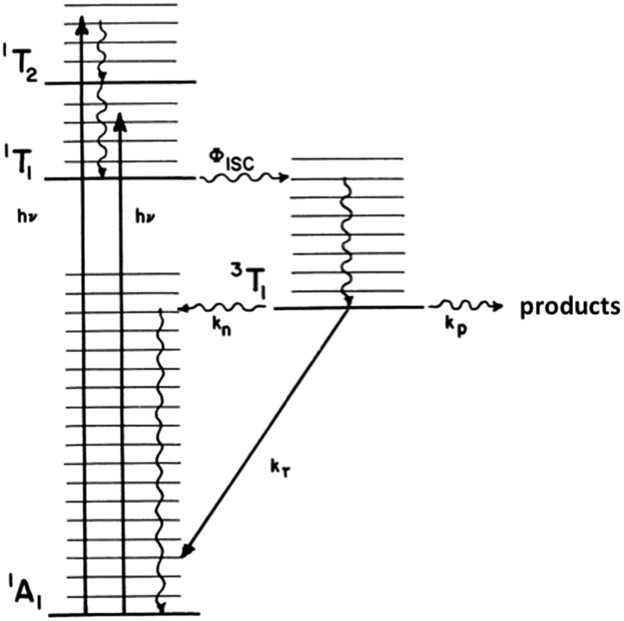
Jablonski excited state diagram for Rh(NH_3_)_6_^3+^ MC states.

For Rh(NH_3_)_6_^3+^, the photosubstitution chemistry (eqn (5), *Φ*_A_ = 0.075 in 296 K aq. solution, A = NH_3_) and the luminescence properties proved to be largely independent of the excitation wavelengths.^18^ These observations, combined with early sensitization experiments,^24^ led to the conclusion that initial LF excitation to form ^1^MC* states was followed by efficient internal conversion/intersystem crossing to the lowest energy ^3^MC* state (*i.e.* the ^3^T_1_)^25^ from which emission or reaction largely occurred (note: *k*_ISC_ values > 5 × 10^9^ s^–1^ have been measured for Rh(iii) ammine complexes).^25^ This progression is envisioned by the Jablonski diagram shown in Fig. 6. Accordingly, one can treat the photoreaction and emission quantum yields in terms of the excited state rate constants as in eqn (3). Thus, for example, *Φ*_A_ = *k*_A_/(*k*_A_ + *k*_n_ + *k*_r_). As seen for the ruthenium complexes, the modest quantum yield for eqn (5) and the very weak emission clearly indicate that the dominant pathway from the ^3^T_1_ is nonradiative deactivation, thus *k*_n_ > *k*_A_ ≫ *k*_r_. If that were the case, then inhibiting *k*_n_ would have the potential to increase *Φ*_A_.5
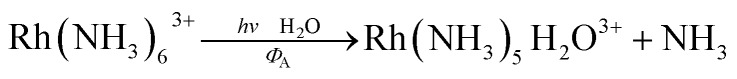



The question was: how to test this idea by perturbing *k*_n_? To do so, we drew upon studies by Glen Crosby and coworkers, who had shown that the perdeuterated complex Rh(ND_3_)_6_^3+^ had dramatically longer luminescence lifetimes in frozen solutions at 77 K than did the perprotio analog.^26^ Since no photochemistry occurred under those conditions and the emission was a minor deactivation pathway, this meant that exchanging the N–H's for N–D's significantly decreased the rate of nonradiative deactivation. This, now a well recognized phenomena, is the result of a weak-coupling mechanism^27^ for nonradiative deactivation that occurs through intramolecular excitation of the highest frequency molecular vibrations (*ν*_NH_ in the case of Rh(NH_3_)_6_^3+^). The question remained whether this effect on *k*_n_ would carry over to studies in fluid solutions. Indeed, we were able to show that exchanging the amines with D_2_O to give Rh(ND_3_)_6_^3+^, led to roughly doubled values of *Φ*_A_ in ambient *T* solutions, consistent with this premise.^19^

However, given that eqn (3) interprets the quantum yield as a ratio of rate constants, the increased *Φ*_A_ is only indirect evidence in support of the conclusion that we were suppressing *k*_n_ partially by perdeuterating the complex. One could instead draw the seemingly less likely conclusion that the amine perdeuteration leads to an increase in the photoreaction rate constant *k*_A_. This conundrum led us to increase efforts to measure excited state lifetimes under conditions directly relevant to the photoreaction pathways. Initially these studies were with Doug Magde at UC San Diego, but this quest was reinforced by access to one of the first ns pulsed Nd/YAG lasers commercially available. We were thus able to measure lifetimes of the faint phosphorescence from the ^3^MC* states of various perprotio and perdeuterio Rh(iii) ammine complexes in fluid aqueous solutions.^21^,^28^

By using eqn (4) and the measured *Φ*_A_ and *τ* values for Rh(NH_3_)_6_^3+^ (0.08 and 21 ns) and Rh(ND_3_)_6_^3+^ (0.15 and 40 ns) in 298 K aq. solution, we calculated ES *k*_A_ values of 3.8 × 10^6^ s^–1^ and 3.3 × 10^6^ s^–1^, respectively, for these two ions.^28^ Obviously, ammine deuteration had only a small effect, and in the wrong direction, on the *k*_A_ values. Similarly, the respective *k*_n_ values were calculated as 4.4 × 10^7^ s^–1^ and 1.9 × 10^7^ s^–1^. These results clearly confirm that the deuterium effect on *Φ*_A_ is entirely due to suppression of the weak-coupling component of the nonradiative deactivation (*k*_n_), not to the acceleration of the excited state rate of ligand dissociation (*k*_A_).

The related chlorido complex Rh(NH_3_)_5_Cl^2+^ provided an opportunity to probe the actual mechanism by which the metal-centered excited state undergoes ligand substitution. Excitation of its ligand field bands leads both to competitive release of an ammine and of a chloride in aq. solution (eqn (6)).^21^ Of the three likely Rh-containing photoproducts, *cis*- and *trans*-Rh(NH_3_)_5_(H_2_O)Cl^2+^ and Rh(NH_3_)_5_H_2_O^3+^, only the latter two were observed. However, isotopic labeling studies by Skibsted demonstrated that the labilized NH_3_ originates roughly equally from axial and equatorial sites, so equatorial NH_3_ labilization must be accompanied by coordination sphere isomerization.^29^6
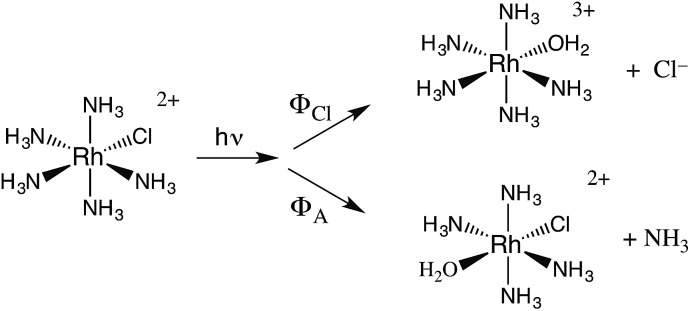



One can rationalize these products from the one-electron excited states predicted by group theory. The lowest energy ^3^T_1_ excited state shown for the octahedral complex in Fig. 6 is split in the *C*_4v_ symmetry of a complex such as Rh(NH_3_)_5_X^2+^ to give ^3^E and ^3^A_2_ states. If X is a weaker σ-donor ligand than NH_3_, then the ^3^E is the LEES. The one electron configuration of that ES places the excited electron in the σ-antibonding (d_*z*^2^_) orbital directed along the unique axis, suggesting that *trans* NH_3_ or Cl^–^ will be labilized. Consistent with this view, Cl^–^ photoaquation is the principal photoreaction. Furthermore, from a statistical perspective, it appears that the axial ammonia is labilized four times as readily as any single equatorial ammonia. However, the occurrence of significant equatorial labilization suggests that the ^3^A state may also play a role.

If X^–^ is a stronger σ-donor than is NH_3_, then the ^3^A_2_ state is the LEES, and the excited electron is primarily in the equatorial σ-antibonding (d_*x*^2^–*y*^2^_) orbital. Labilization would then be expected along the axes perpendicular to the principal axis. Consistent with this argument, the cyanido complex undergoes NH_3_ labilization of the equatorial ammine to give *cis*-Rh(NH_3_)_4_(H_2_O)CN^2+^ with a *Φ*_NH_3__ of 0.09 in ambient temperature aqueous solution (eqn (7)).^30^ Furthermore, selective isotopic labeling demonstrated that the labilized NH_3_ originates from the equatorial sites as predicted.^31^7
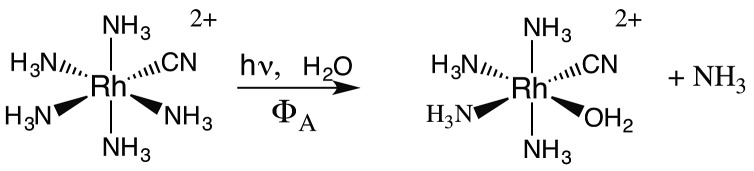



The quantum yields for eqn (6) are *Φ*_A_ = 0.02 and *Φ*_Cl_ = 0.18 in 298 K aq. solution.^21^ We measured the emission quantum yield *Φ*_r_ for the ^3^MC* state of Rh(NH_3_)_5_Cl^2+^ as 3 × 10^–5^ under these conditions,^21^ so this compound also represents another case where nonradiative decay is the principal deactivation pathway from the ^3^MC* state. Accordingly, the emission lifetime (35 ns) of the perdeuterio complex Rh(ND_3_)_5_Cl^2+^ is significantly longer than that (14 ns) of Rh(NH_3_)_5_Cl^2+^. By using eqn (4), the respective excited state rate constants for the latter ion calculated from the quantum yields and lifetimes are *k*_Cl_ = 1.4 × 10^7^ s^–1^, *k*_A_ = 0.14 × 10^7^ s^–1^, *k*_r_ = 2.3 × 10^3^ s^–1^ and *k*_n_ = 5.6 × 10^7^ s^–1^. By measuring the quantum yields and lifetimes at several temperatures, we calculated an activation energy (*E*_a_) of 25 kJ mol^–1^ for *k*_Cl_. This result clearly indicates that the potential well for the reactive ^3^MC* state of Rh(NH_3_)_5_Cl^2+^ is well defined. In other words, it is a “bound” state as described in Fig. 4.

For comparison, heating an acidic, aqueous solution of Rh(NH_3_)_5_Cl^2+^ leads to Cl^–^ aquation only.^32^ This displays a first-order rate law, and extrapolation to ambient temperature gives *k*′_Cl_ = 5.4 × 10^–8^ s^–1^ for the ground state reaction. Thus, the ^3^E triplet ES is >14 orders of magnitude more reactive toward chloride labilization than is the ground state. Furthermore, the *E*_a_ (120 kJ mol^–1^) for *k*′_Cl_ is much higher than that for the analogous photoreaction.

These comparisons do not differentiate the possible “unimolecular” mechanisms by which the ^3^MC* state might undergo cleavage of the metal–ligand bonds. For example, is this reaction the result of a dissociative substitution mechanism, where M–L bond breaking is the predominant contribution to the activation energy of that step, as might be implied by the LEES (t_2g_)^5^(e_g_)^1^ electronic configuration for a hexaammine complex? Alternatively, the t_2g_ vacancy (relative to the (t_2g_)^6^ ground state) might enhance a more associative pathway. We explored this by determining the ES reaction rate constants *k*_Cl_ and *k*_A_ in different solvents.^33^ The key result was that changing the solvent polarity (and nucleophilic character) had a modest impact on *k*_A_ but major influence on *k*_Cl_, reducing the latter dramatically in the less polar solvents. This argues against a major associative component to the exchange of the coordinated NH_3_ with the solvent molecule. While it could suggest such a component to the Cl^–^ labilization, a more likely explanation is that the barrier to dissociation is strongly affected by the solvation of the developing charge separation as the (NH_3_)_5_Rh^3+^···Cl^–^ bond dissociates.

In this context, a collaborative investigation with Rudi van Eldik^23^ measured the photoreaction quantum yields and lifetimes for aq. Rh(NH_3_)_5_Cl^2+^ as a function of hydrostatic pressure up to 200 MPa, and activation volumes Δ*V*‡*i* were determined. These results are self-consistent with the solvent effects on the analogous reactions in the context that the solvation of the developing charge separation as Cl^–^ dissociates from the Rh(iii) center has a major impact on the barrier for the *k*_Cl_ pathway. Although no experiment proves a mechanism, these results are clearly consistent dissociative labilization mechanisms from the ^3^MC* state.

## Bimolecular processes

Our discussion has so far focused on unimolecular processes that occur subsequent to excitation. For transition metal complexes these processes will be dominated by the competing nonradiative deactivation, emission and “unimolecular” events such as ligand solvolysis, isomerization or redox reactions like eqn (8) (TPP^2–^ = tetraphenyl-porphyrinato dianion).^34^ However, excited state complexes may also participate in bimolecular events such as energy or electron transfer to another chromophore.8
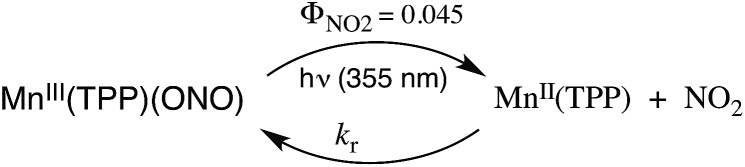



The simplest case is one in which there is a single state or set of states in thermal equilibrium that is largely responsible for the photochemical and photophysical behavior of the metal complex. Such is the case for the extensively investigated Ru(bpy)_3_^2+^ ion.^12^ For this species, the situation described in Fig. 3 is reversed, *i.e.*, the lowest energy ES is the ^3^MLCT ES, which is relatively unreactive toward ligand substitution. As a consequence, Ru(bpy)_3_^2+^ salts are strongly luminescent even in ambient temperature solutions (*Φ*_em_ = 0.063 in 298 K deaerated H_2_O, 0.040 in aerated H_2_O),^35^ although the major decay pathway remains the non-radiative deactivation which is largely attributed to the back population from ^3^MLCT state to the lowest energy ^3^MC state. The roughly microsecond lifetimes and the sensitivity of luminescence detection make such a species ideal for probing bimolecular quenching mechanisms either by energy transfer or by electron transfer. Notably, excited states are both stronger oxidants and stronger reductants than the respective ground state, and this feature has been extensively exploited in applications such as solar energy conversion,^36^,^37^ organic synthesis,^38^ and new photo-initiated cancer drugs.^39^

From a kinetics perspective, quenching may have a major impact on the luminescent lifetime *τ*. The quenching rate constants *k*_q_ in solution can be determined by measuring *τ* as a function of quencher concentrations [Q] using Stern–Volmer plots of *τ*_0_/*τ vs.* [Q] according to eqn (9) where *τ*_0_ is the lifetime in the absence of quencher. The value of *k*_q_ will depend on the viscosity of the solvent (hence the rate of diffusion) and the operating quenching mechanism(s). If the latter involves energy transfer, the rate will depend on the spectral overlap between the donor and acceptor pair as well as the energies of the relevant excited states. If it involves electron transfer, then the key issue will be the relative redox potentials of the excited state species and of the quencher. We illustrate these points with photo-luminescent Cu(i) complexes.

9 *τ*_0_/*τ* = 1 + *k*_q_*τ*_0_[Q]

### The Cu(i) cluster Cu_4_I_4_py_4_

Luminescence in first row transition metal complexes is frequently bypassed by the presence of lowest energy MC states that are too short-lived to have measurable emissions. Thus, filled shell d^10^ metal systems offer an opportunity to observe other excited states.^40^ Excellent examples are the Cu(i) complexes of the type Cu(DMP)_2_^+^ ion (DMP = 2,9-dimethyl-1,10-phenanthroline) described by McMillin *et al.*40a,b In the absence of MC* states these moieties display MLCT emissions. Here we shall focus on the photophysical/chemical behavior of a different type of Cu(i) complexes, tetranuclear clusters such as Cu_4_I_4_py_4_ that consist of Cu_4_ tetrahedra with short Cu–Cu distances. Bridging iodides and terminal pyridine ligands complete the unit. Related polynuclear copper(i) clusters have drawn recent interest for potential applications in OLEDs.^41^
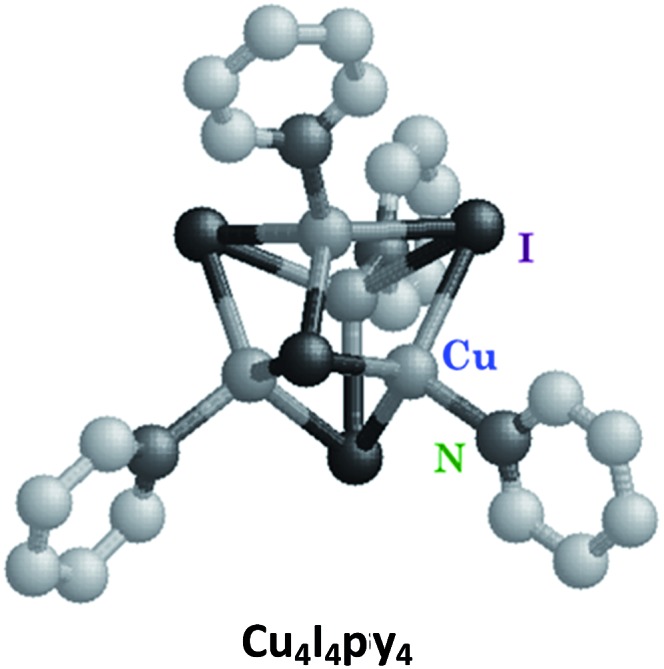


**Fig. 7:**
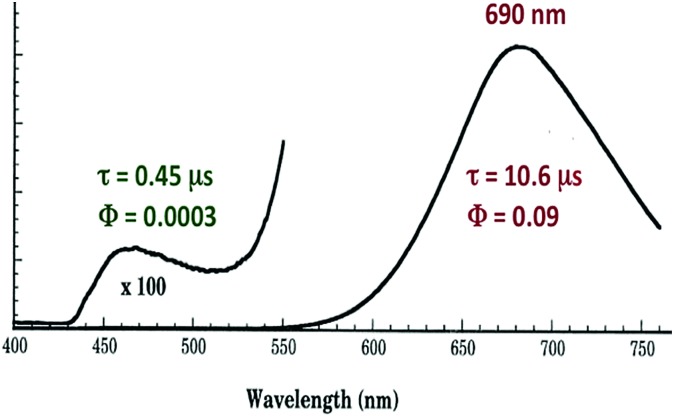
Emission spectrum from Cu_4_I_4_py_4_ in 296 K toluene solution. The excitation wavelength was 350 nm.

The photophysical properties of Cu_4_I_4_py_4_ demonstrate marked environmental sensitivity, *i.e.*, the luminescence spectra change reversibly by varying the temperature and the rigidity of the medium^42^ owing to the presence of multiple emitting excited states. In ambient solutions, Cu_4_I_4_py_4_ displays two distinct emission bands that also demonstrate different lifetimes (Fig. 7). This behavior is in obvious contrast to the patterns seen for the Rh(NH_3_)_6_^3+^ and Ru(bpy)_3_^2+^ ions described above, where single emissions from lowest energy MC and MLCT states were respectively recorded.

The strong, longer-lived (*τ* = 10.6 μs) lower energy (LE) emission (*λ*_max_ = 690 nm) from Cu_4_I_4_py_4_ in 296 K toluene solution was assigned to a triplet cluster-centered (^3^CC) ES, resulting from a combination of delocalized iodide-to-copper charge transfer and d–s transitions.^42^ The weaker, shorter-lived (*τ* = 0.45 μs) higher energy (HE) band (*λ*_max_ = 460 nm) was assigned to a triplet halide-to-ligand (pyridine) charge transfer (^3^XLCT) ES.

**Fig. 8:**
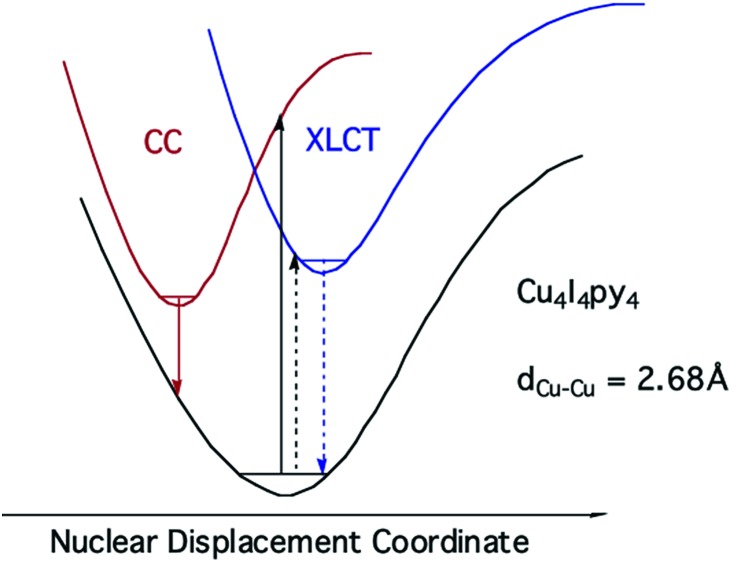
ES diagram to explain multiple emissions from Cu_4_I_4_py_4_.

Notably, when the pyridines are replaced by the aliphatic amine piperidine, the LE band is still present, but the HE band is not.^43^ Relative to the GS, the ^3^CC and ^3^XLCT ES of Cu_4_I_4_py_4_ are distorted along different trajectories, since the former ES is formed by promoting an electron from filled orbitals that are non-bonding (or even anti-bonding) to orbitals that are bonding with respect to the Cu_4_ cluster. As a result the surfaces of the ^3^CC and ^3^XLCT states overlap poorly, and there is an energy barrier for internal conversion from the ^3^XLCT state to the lower energy ^3^CC excited state (Fig. 8). This is especially apparent at lower temperatures where the ^3^XLCT emission is much stronger. Computational studies clearly support these assignments.^44^

**Scheme 1:**
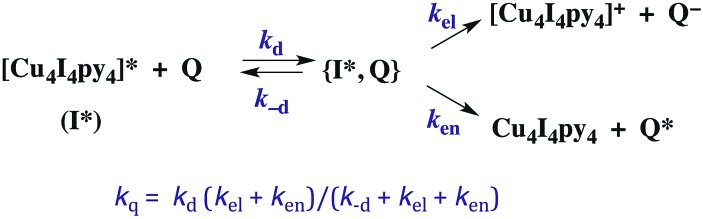
Competitive energy transfer and electron transfer quenching of the ^3^CC excited state of Cu_4_I_4_py_4._

The kinetics of biomolecular quenching of the Cu_4_I_4_py_4_^3^CC emission were explored in dichloromethane solution using nitrobenzenes and ferricenium cations as well as a series of tris((β-dionato))chromium(iii) derivatives CrL_3_.^45^ The CrL_3_ complexes quench by competitive energy transfer and electron transfer mechanisms (Scheme 1). By varying L, one is able to markedly change the reduction potential of the CrL_3_ species, but the energy (∼1.3 μm^–1^) of the lowest ES of this species, a Cr(iii) centered ^2^MC state, is essentially unchanged.^46^

**Fig. 9:**
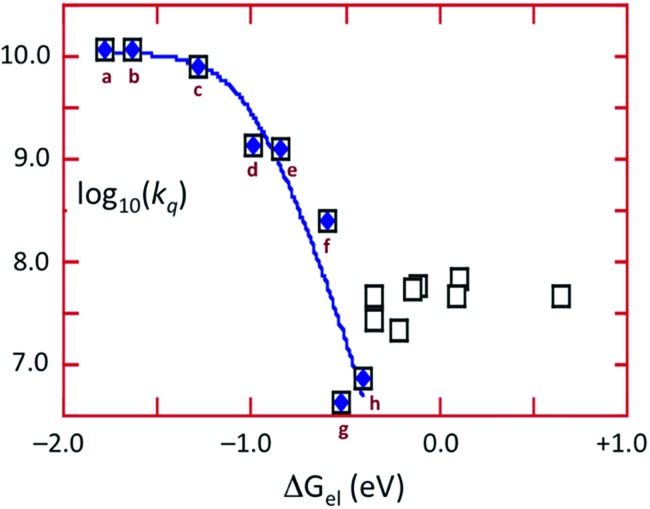
Plot of log *k*_q_*vs.* Δ*G*oel for the quenching of emission from Cu_4_I_4_py_4_* by oxidants. The curve represents the best fit according to the Marcus model for electron transfer quenching by (a) Fc^+^, (b) Me_2_Fc^+^, (c) Me_10_Fc^+^, (d) Cr(hfac)_3_ (hfac = hexafluoroacetyl-acetonato), (e) 1,4-benzoquinone, (f) *p*-dinitrobenzene, (g) *o*-dinitrobenzene, (h) *m*-dinitrobenzene. Open squares represent CrL_3_ complexes considered to quench largely by energy transfer.

The energy of the ^3^CC state is ∼1.66 μm^–1^ (2.06 V) and the excited state reduction potential *E*_1/2_([Cu_4_I_4_py_4_]^+^/[Cu_4_I_4_py_4_]*) is ∼–1.78 V (*vs.* the ferricenium/ferrocene couple). Thus the ^3^CC state is a very powerful reducing agent, but it also is capable of undergoing energy transfer quenching by the CrL_3_ centers. The bimolecular rate constant for the latter process was shown to be ∼10^7.9^ M^–1^ s^–1^, while contributions to the quenching by an electron-transfer mechanism were evident for those *Q* with reduction potentials *E*_1/2_(*Q*/*Q*^–^) less than 1.4 V. This behavior is demonstrated by the plot of log_10_(*k*_q_) *vs.* the Δ*G*^o^ of the electron transfer quenching step calculated according to eqn (10) (Fig. 9). The curve represents the fit to the Marcus model^47^,^48^ for the quenching rate constants from *Q* for which electron transfer processes are expected. The substantial over-potential required before electron transfer quenching becomes dominant can be attributed to a large contribution to the inner-sphere reorganization energy owing to the distortion of the cluster centered excited state.

10 Δ*G*oel = –{*E*_1/2_(*Q*/*Q*^–^) – *E*_1/2_([Cu_4_I_4_py_4_]^+^/[Cu_4_I_4_py_4_]*)}

## Flash photolysis probes of reactive intermediates

**Fig. 10:**
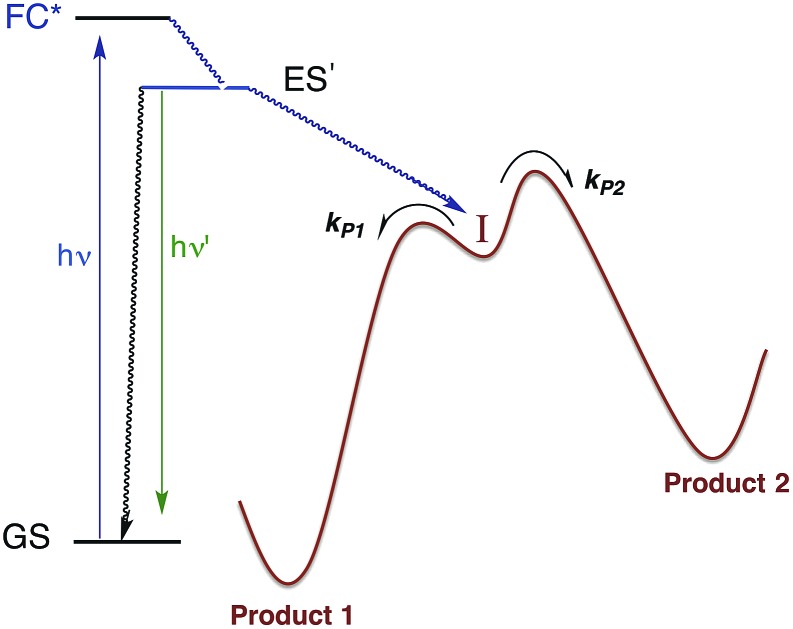
Illustration of reactive intermediate formation as the primary photoproduct from excitation into the Franck–Condon state. **I** can react by competitive pathways to form more than one product.

Within the anatomy of a photoreaction, there are potentially a number of steps between the initial ground state and the final product(s) as qualitatively illustrated in Fig. 10. The first is the initial absorption of light that prepares a Franck–Condon state (FC*) that is both electronically and vibrationally excited. The FC* may undergo reaction if oriented along a particular trajectory, or it may undergo vibronic and electronic decay *via* internal conversion to lower energy states of the same spin multiplicity and/or intersystem crossing to lower energy ES of different multiplicity(ies). From either type of ES, luminescence (fluorescence or phosphorescence, respectively), non-radiative deactivation to the GS and/or chemical reaction can occur. The focus here will be on the reactive pathway, since primary photoproduct(s) formed, while no longer electronically excited, may yet be quite reactive under the experimental conditions. Thus, the final products observed may result from secondary pathways of the intermediate(s) **I**.

There are several ways to probe such intermediates. For example, in low temperature matrices, the activation energies for surmounting the barriers illustrated in Fig. 10 may be sufficient to inhibit further reaction. In that case, photolysis can be used to generate **I**, which then can be characterized by conventional spectroscopy. An alternative (and complementary) approach is to use flash photolysis to generate such species and fast detection techniques to characterize their spectroscopy and reaction dynamics as they evolve to the final products.

Our first flash photolysis apparatus was a “conventional” flash lamp system with a design not altogether that different from that first built by Norrish and Porter, who were awarded the 1966 Nobel Prize in Chemistry for their contributions to fast reaction kinetics. This system operated by discharging hundreds of Joules of electrical energy from a capacitor bank through 10–20 cm long cylindrical quartz flashlamps filled with xenon gas. The white light flash lasted about 10 μs and irradiated a sample cell with a path of similar length. If one were sufficiently careful to avoid saturating the PMT detector with scattered light, it was possible to record flash-induced temporal changes in the transient spectra.

### Flash photolysis of Ru(NH_3_)_5_(py)^2+^

**Scheme 2:**
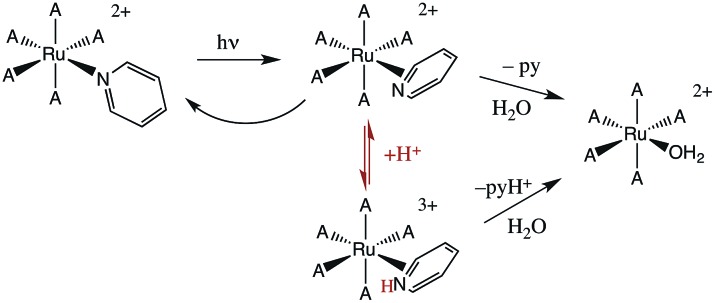
Possible scenario for flash photolysis dynamics of Ru(NH_3_)_5_(py)^2+^ (A = NH_3_).

The first system that we used this flash photolysis apparatus to study was the highly colored Ru(NH_3_)_5_(py)^2+^ ion that had first stirred our interest in photochemistry. Our early flash studies indicated the formation of a transient species that partially returned to the starting material on a millisecond time scale.^49^ In acidic solution, the recovery was slower and less of the starting ion reformed. These observations paralleled our finding that pyridine photoaquation quantum yields are higher at lower pH.^8^ On these bases, we proposed that pyridine aquation occurs *via* formation of a transient species by which the Ru–py bonding has isomerized from monodentate coordination to a π-complex such as the η^2^-py species illustrated in Scheme 2. It is notable that a recent study9*b* using laser flash photolysis techniques and DFT computations confirmed the formation of such an intermediate, although Ru(ii) coordination at a pyridine C–C bond was concluded to be the most stable η^2^-pyridine complex.

Flash photolysis also provides a valuable tool for probing the mechanisms of thermal reactions where the presence of a key reactive intermediate is suspected. We will give several examples to illustrate this approach.

### Wilkinson's catalyst

**Scheme 3:**
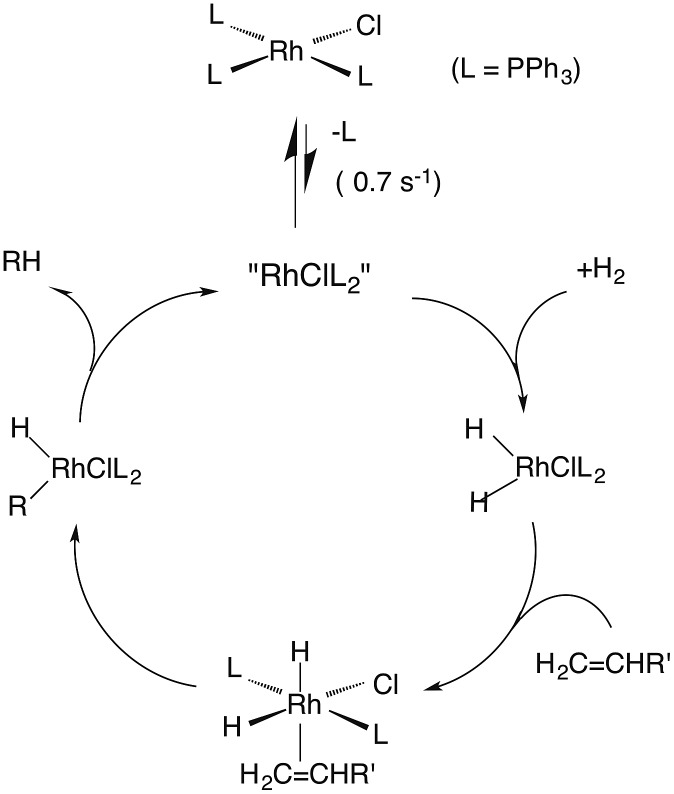
Proposed mechanism for hydrogenation of alkenes by Wilkinson's catalyst (L = PPh_3_).

**Scheme 4:**
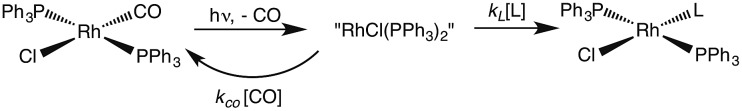
Photodissociation of CO from *trans*-Rh(PPh_3_)_2_(CO)Cl to give the “tricoordinate” intermediate of Wilkinson's catalyst.

One such study was concerned with the reactivity of intermediates proposed in the homogeneous hydrogenation of olefins by the rhodium(i) species Rh(PPh_3_)_3_Cl, first reported by Wilkinson and coworkers.^50^ Those workers and Halpern^51^ had suggested that the key intermediate in the catalytic cycle was the “three coordinate” species Rh(PPh_3_)_2_Cl formed by spontaneous dissociation of PPh_3_ (Scheme 3). One way to access this intermediate is by flash photolysis induced CO dissociation from the carbonyl analog *trans*-Rh(PPh_3_)_2_(CO)Cl (Scheme 4). We studied this reaction first with the conventional flash system,^52^ then later using laser flash photolysis.^53^ In benzene solution, this substrate undergoes reversible photo-dissociation of CO (Scheme 4, *k*_CO_ = 7.8 × 10^8^ M^–1^ s^–1^). By carrying out the experiment in the presence of reactants such as PPh_3_, H_2_ and ethylene, it was possible to determine other second order rate constants for trapping the Rh(PPh_3_)_2_Cl intermediate.

For example, the *k*_L_ for the reaction with PPh_3_ was determined to be ∼6 × 10^7^ M^–1^ s^–1^ in 296 K benzene while that with H_2_ was nearly a factor of 30 slower. In addition, *k*_CO_ values in cyclohexane and dichloromethane were ∼2-fold and 5-fold lower, respectively, suggesting that the intermediate is not three-coordinate, but is weakly bound by solvent in the position vacated by CO.^53^

### Metal carbonyls and the migratory insertion reaction

While flash induced changes in the optical spectra are often particularly valuable for following the dynamics of excited states and reactive intermediates, the interpretable structural information from the transient spectra is often limited by the broadening of the absorption and emission bands in solution. In this regard, other time-resolved spectroscopic techniques can provide new dimensions. Time-resolved infrared (TRIR) spectroscopy is especially effective in probing fast reactions of ES and intermediates generated by the photo excitation of metal carbonyls.^54^–^58^ These compounds display strong IR absorption bands, the positions of which provide insight into the electronic nature of the metal center in the transient species. In order to access such information we constructed a TRIR apparatus that (initially) used a XeCl Excimer laser as the pulsed excitation source, tunable lead salt IR diodes as the detection source and a Hg/Cd/Te solid state fast response IR detector.^59^ This allowed pump-probe experiments on the ns to μs timescale with detection in the *ν*_CO_ region of the IR spectrum.

**Fig. 11:**
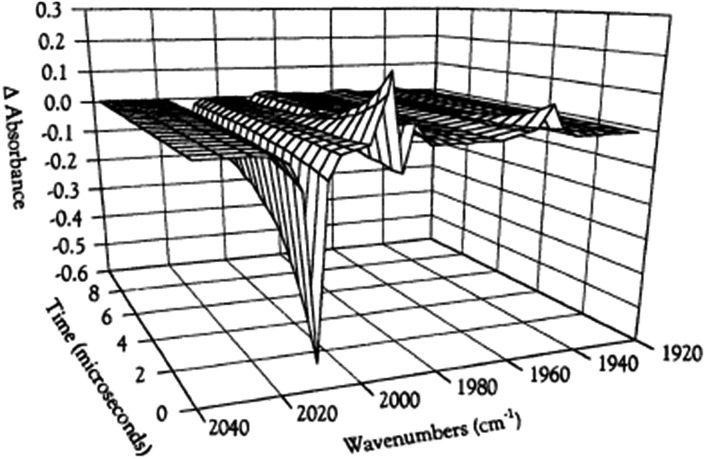
TRIR spectral data resulting form the 308 nm flash photolysis of CH_3_Mn(CO)_5_ in 295 K cyclohexane under 0.1 atm CO.

An example is shown in Fig. 11, which describes the temporal IR difference spectra in the *ν*_CO_ region upon 308 nm flash photolysis of CH_3_Mn(CO)_5_ in cyclohexane solution.^60^ The IR spectrum can be interpreted in terms of CO photodissociation from this entity to give primarily *cis*-CH_3_Mn(CO)_4_(Sol) (eqn (11), Sol being the solvent). Homolytic fragmentation of the Mn–CH_3_ bond was a minor (<10%) photochemical pathway as well.11
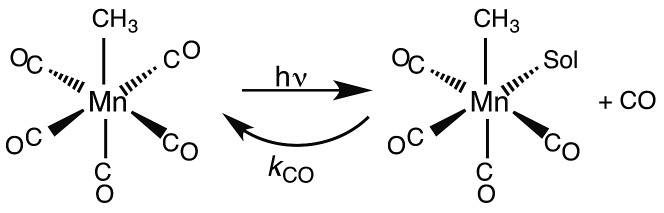



As other studies have shown, the empty site generated by CO photodissociation is often filled by coordination to a solvent molecule, even ones as weakly binding as a saturated hydrocarbon.^61^,^62^ In the presence of excess CO this transient undergoes exponential decay back to CH_3_Mn(CO)_5_ at rates linearly dependent on [CO] and strongly affected by the nature of the solvent. In cyclohexane, the second order rate constant *k*_CO_ (295 K) was 4.5 × 10^8^ M^–1^ s^–1^, while the value in THF (1.4 × 10^2^ M^–1^ s^–1^) was more than 6 orders of magnitude smaller. For reactions in THF, the temperature dependence of *k*_CO_ was investigated over the range 232–293 K, and an Eyring plot gave ΔH* = 76 kJ mol^–l^ and ΔS* = 59 J mol^–l^ K^–l^.^60^ Although these data do not clearly differentiate between associative and dissociative pathways, the positive ΔS* value suggests that considerable Mn–Sol bond breaking is occurring in the rate-limiting step of this substitution pathway.

Another reaction of interest from the catalysis perspective is the “migratory insertion” of carbon monoxide into metal alkyl bonds (eqn (12)). This is the key carbon–carbon bond formation pathway in catalytic carbonylations such as acetic acid synthesis from methanol and alkene hydroformylation.^63^ Alkyl manganese carbonyl complexes such as CH_3_Mn(CO)_5_ were extensively probed as mechanistic models for this important class of organometallic reactions.^64^ Those studies suggest that alkyl migration to a *cis* carbonyl leads to a reactive intermediate in a step promoted by more polar solvents.12
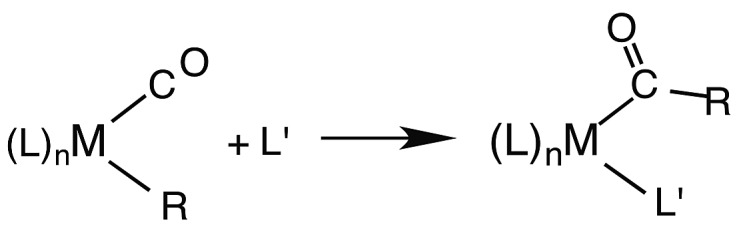



Our strategy for characterizing the structure and reactivity of such intermediates started with the acyl complex. Photodissociation of a ligand L′ from the acyl complex M-C(O)R prepares a reactive species **I** (eqn (13)) with the same composition as the intermediate proposed for migratory insertion. Time-resolved optical and IR spectral studies were then used to interrogate **I** and the dynamics of the reactions with various L′ to give the stable acyl products and of reverse alkyl migration to give the metal alkyl complex M–R.13
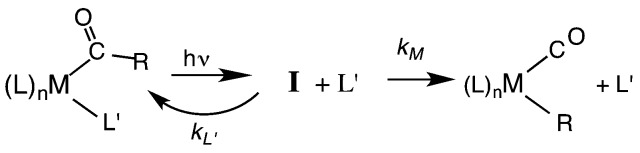



**Fig. 12:**
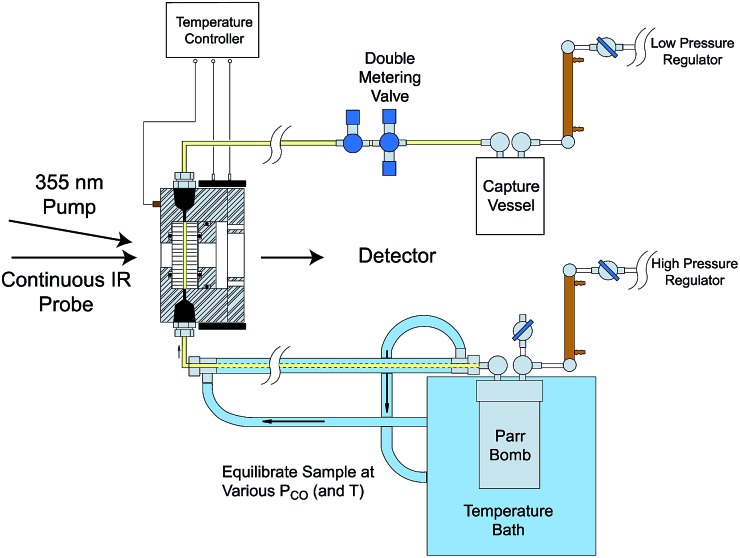
HP/VT flow cell for TRIR studies at higher pressures (up to 100 atm) and temperature (up to 150 °C). Note that the transit of solution through the cell was sufficiently fast that under the repetitive pulsing of the 355 nm pump (3rd harmonic of a Nd:YAG ns laser) solutions are refreshed between pulses (figure adapted from ref. 70).

Comparisons of rates measured for **I** in this manner to the competitive reactivities deduced for intermediates in the thermal migratory insertion kinetics provide guidance relevant to mechanisms of these reactions. We studied such migratory insertion pathways by starting with Mn(CO)_5_(C(O)R),^65^,^66^ (η^5^-C_5_H_5_)Fe(CO)(C(O)R),^67^ CH_3_C(O)Co(CO)_3_PPh_3_ (ref. 68–70) and [CH_3_Ir(CO)_2_I_3_]^–^.^71^ Notably, the latter two systems are relevant to industrial catalysts for the hydroformylation of higher molecular weight alkenes (Shell catalyst)^64^ and for methanol carbonylation to acetic acid (Cativa catalyst).^72^ For the latter two studies, it was necessary to build a high pressure/variable temperature (HP/VT) TRIR sample flow cell (Fig. 12)^70^ so that conditions approaching those of the industrial catalysts could be used. A benefit of this system was precise control of reaction temperatures in order to determine activation parameters.

Homogeneous cobalt carbonyl catalysts for alkene hydroformylation have been in use since their discovery in 1938 by Otto Roelen at Ruhrchemie AG. The original catalyst was based on simple cobalt carbonyl precursors; however, phosphine modified catalysts provide more favorable linear-to-branched selectivity and greater stability at higher temperatures. A key intermediate relevant to such catalysts is the “unsaturated” acyl complex RC(O)Co(CO)_2_L (**I_Co_**).^73^ This intermediate may be stabilized by as a η^2^-acyl structure,^74^ a view that has been supported by DFT calculations.^75^

We used flash photolysis of the phosphine modified cobalt acyl complex CH_3_C(O)Co(CO)_3_PPh_3_ (**A_Co_**) to probe the TRIR spectra and reactivity of the unsaturated intermediate **I_Co_**.^68^–^70^ The IR spectrum of **A_Co_** shows the weak A_1_ and strong E *ν*_CO_ bands expected for the local C_3V_ symmetry. Events following 355 nm photolysis of **A_Co_** are indicated by the temporal TRIR spectra. Readily apparent changes are the prompt bleach of the *ν*_CO_ bands characteristic of **A_Co_**, and the prompt formation and decay of a transient species **I_Co_** with strong absorbances at 1915 and 1947 cm^–1^ followed by growth of a photoproduct that was shown to be CH_3_Co(CO)_3_PPh_3_ (**M_Co_**).

**Scheme 5:**
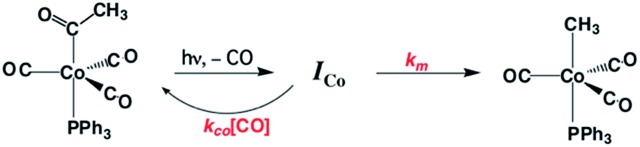
Photolysis of CH_3_C(O)Co(CO)_3_(PPh_3_).

**Scheme 6:**
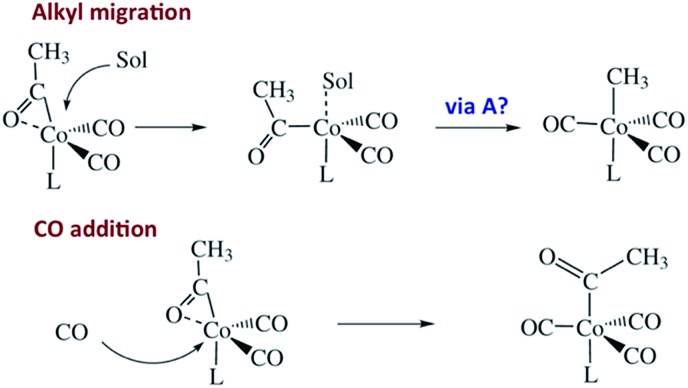
Potential mechanisms for the decay of **I_Co_** to **M_Co_** and **A_Co_**.

Regeneration of **A_Co_** is linearly dependent on [CO] while formation of **M_Co_** was independent of [CO] (*k*_obs_ = *k*_CO_[CO] + *k*_M_; *k*_CO_ = 1.14 × 10^7^ M^–1^ s^–1^; *k*_M_ = 6.2 × 10^4^ s^–1^ in 298 K benzene-d_6_) in accord with the scenario illustrated in Scheme 5. Although we will not detail the arguments here, solvent and deuterium isotope effects on *k*_CO_ and *k*_M_ indicate that the η^2^-acyl is indeed the most likely structure for **I_Co_**.^68^Scheme 6 offers proposed mechanisms for the steps depleting **I_Co_**. The solvent insensitive *k*_CO_ has a very small Δ*H*^‡^ (6 kJ mol^–1^) but a sizable, negative Δ*S*^‡^ (–92 J mol^–1^ K^–1^), and these suggest an associative mechanism to regenerate **A_Co_**. The *k*_M_ rates to give **M_Co_** are accelerated by donor solvents and reflect more intimate involvement of solvent in the methyl migration. This may be necessary owing to the orientation of the alkyl group in **I_Co_** that is not well suited for migration to the metal center.

### Reaction of the bioregulatory molecule NO with heme iron

Nitric oxide plays major roles in cardiovascular regulation, and one key bioregulatory target is the ferroheme protein soluble guanylyl cyclase (sGC), which displays a remarkable specificity for NO.^76^ In this context we used flash photolysis to study the rates and mechanisms of NO reactions with biologically relevant metal centers.^77^,^78^ This approach was not unique to us, but was used by Hoffman and Gibson,^79^ Magde,^80^ Olson,^81^ and others.^82^ to investigate the kinetics of various heme centers with NO. However, until our studies,^83^ there had been few systematic mechanistic investigations of metal–NO bond formation.

One precedent is the report by Taube and coworkers^85^ of the nitrosylation kinetics for the Ru(iii) complex Ru(NH_3_)_6_^3+^ in acidic aqueous solution (eqn (14)). They measured a rate constant (*k*_NO_ = 0.2 M^–1^ s^–1^ at 298 K), which was much larger than that for NH_3_ substitution by other ligands. They concluded that the reaction with NO must be associative, whereby the low spin d^5^ Ru(iii) center engages the odd electron of NO to give a 7-coordinate Ru(NH_3_)_6_(NO)^3+^ transient. Subsequent activation parameter measurements gave a relatively small Δ*H*^‡^ (41 kJ mol^–1^) and a large and negative Δ*S*^‡^ (–114 J K^–1^ mol^–1^)^86^ as well as a negative activation volume Δ*V*^‡^ (–14 cm^3^ mol^–1^)^87^ consistent with this view.

14 Ru(NH_3_)_6_^3+^ + NO + H^+^ → Ru(NH_3_)_5_(NO)^3+^ + NH_4_^+^

Should a similar mechanism be valid for the high spin d^5^ Fe(iii) porphyrinato complexes? To probe this question, we investigated the flash photolysis kinetics of aqueous solutions containing Fe^III^(TPPS) (TPPS = tetra(4-sulfonato-phenyl)porphyrinato) and NO, which are in labile thermal equilibrium with *k*_11_ = 10^3^ M^–1^ (298 K).^77^,^83^ Flash photolysis leads to NO photolabilization from Fe^III^(TPPS)(H_2_O)(NO), followed by a relaxation of the system back to equilibrium (eqn (15)). Under excess NO, the latter process is exponential. Linear plots of the resulting *k*_obs_ values *vs.* [NO] follow the relationship *k*_obs_ = *k*_off_ + *k*_on_[NO] from which the values of *k*_off_ = 0.5 × 10^3^ s^–1^ and *k*_on_ = 4.5 × 10^5^ M^–1^ s^–1^ (298 K) were determined.15
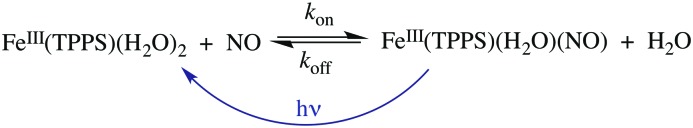


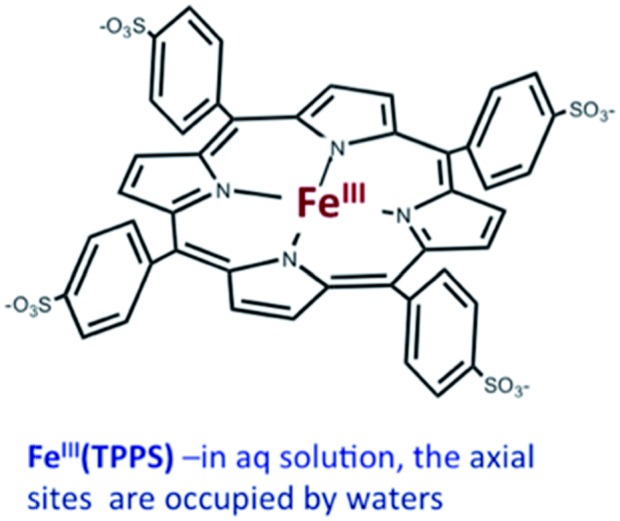
The temperature dependence of these rate constants over the range 298–318 K, gave the Δ*H*^‡^ and Δ*S*^‡^ values for the NO “on” and “off” pathways of Fe^III^(TPPS) as 69 kJ mol^–1^ and +95 J mol^–1^ K^–1^ for *k*_on_ and 76 kJ mol^–1^ and 60 J mol^–1^ K^–1^ for *k*_off_, respectively.^83^

**Fig. 13:**
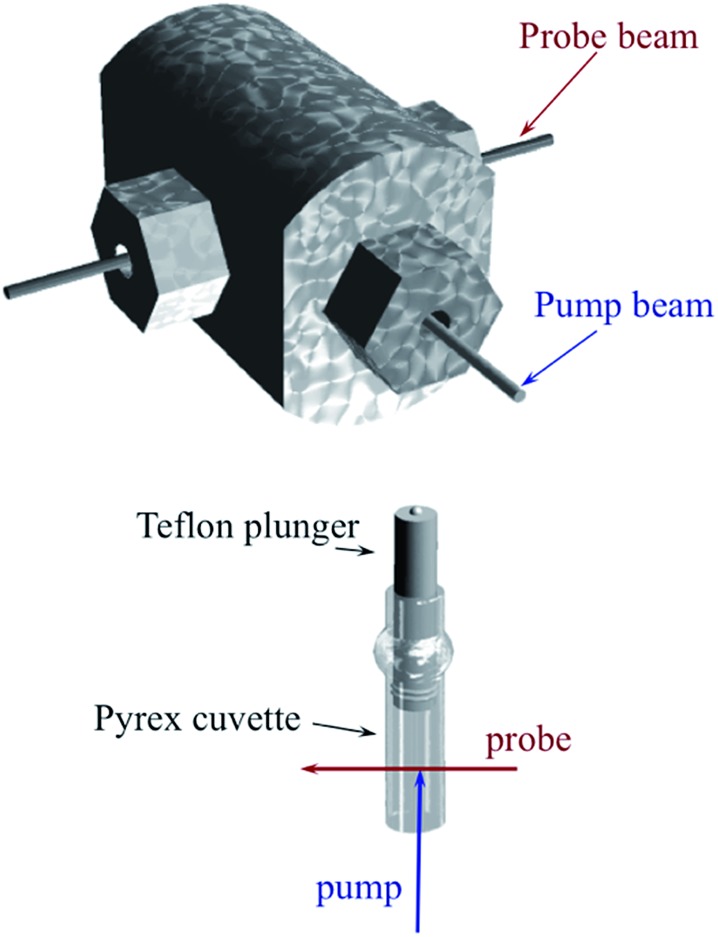
Illustration of cell for measuring the effects of hydrostatic pressures on the dynamics of excited states or intermediates generated by laser flash photolysis (pump) as detected using UV or visible light (probe). The Pyrex or quartz cuvette that is inserted into the HP cell has a sealed Teflon plunger that transmits the applied pressure to the solution. The pump and probe light are transmitted into (and out of) the HP cell through sapphire windows strong enough to withstand the applied pressure.

Another parameter is the activation volume Δ*V*^‡^, which reflects the sensitivity of the rate constants to pressure (Δ*V*^‡^ = –*RT*(d(ln *k*))/d*P*_hyd_)_T_, where *P*_hyd_ is the applied hydrostatic pressure. Pressure effects on laser flash photolysis kinetics were measured in these laboratories using the laser flash photolysis cell illustrated in Fig. 13, which was designed to function from 0.1 to 400 MPa. The experiment involved measuring *k*_obs_ over a range of [NO] to determine *k*_on_ and *k*_off_ for each hydrostatic pressure investigated. Plots of ln(*k*_on_) and of ln(*k*_off_) *vs.* P were linear in this pressure region and from these Δ*V*‡on = +9 cm^3^ mol^–1^ and Δ*V*‡off = +18 cm^3^ mol^–1^ were determined.^83^

These activation parameters suggest the reaction pathways envisioned in eqn (16) and (17). This pathway also predicts that Fe^III^(TPPS)(H_2_O)_2_ will exchange its coordinated waters with solvent much faster than the reaction with NO, which was present at mM concentrations. This indeed is the case; H_2_O exchange with Fe^III^(TPPS)(H_2_O)_2_ occurs with a reported first order rate constant (*k*_ex_ = 1.4 × 10^7^ s^–1^ in 298 K water)^88^ far exceeding the *k*_obs_ values determined at any [NO].16
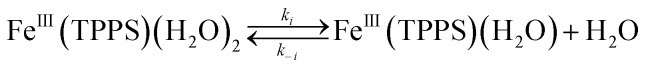

17
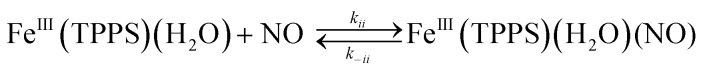



A more recent reexamination of the exchange reaction^89^ using variable temperature/pressure NMR techniques reported the values Δ*H*‡ex = 67 kJ mol^–1^, Δ*S*‡ex = +99 J mol^–1^ K^–1^ and Δ*V*‡ex = +7.9 cm^3^ mol^–1^, which are in remarkable agreement with the activation parameters measured by flash photolysis for the *k*_on_ pathway with NO.^83^ The same factors that determine the NO reaction with Fe^III^(TPPS)(H_2_O)_2_ therefore dominate solvent exchange in agreement with a dissociative mechanism, the limiting step being eqn (16). Not surprisingly, the substitution mechanism for the labile, high spin d^5^ Fe^III^(TPPS)(H_2_O)_2_ complex is markedly different from the associative mechanism described above for the low spin d^5^ Ru(NH_3_)_6_^3+^ ion.

Studies with met-myoglobin came to similar mechanistic conclusions for the reactions of this ferri-heme protein with NO.^84^ In contrast, a flash photolysis study of the NO reaction with the ferrous analog Fe^II^(TPPS) gave the much larger value of *k*_on_ = 1.5 × 10^9^ M^–1^ s^–1^ (298 K), and a value of *k*_off_ so small (6.4 × 10^–4^ s^–1^) that it had to be measured by a different technique.^83^ Furthermore, the *k*_on_ value for the ferrous complex is much less temperature sensitive (Δ*H*‡on = 24 kJ mol^–1^) consistent with a reaction pathway having a rate approaching the diffusion limit in aqueous solution. Analogously large *k*_on_ values have also been reported for the ferrous heme protein sGC.^90^ Since activating this protein is a key process in the endogenous control of blood pressure by NO, it is obvious that the reaction must be very fast, as endogenous NO concentrations involved are very low.^91^

## Applying what we know and learning new tricks: photochemical uncaging of nitric oxide and other bioactive molecules

In many fundamental investigations, there is often later recognition that the knowledge gained can be utilized for practical applications not perceived at the onset. This is especially true in chemistry, a discipline that lies at the interface between truly fundamental science and the chemical, materials and pharmaceutical industries. Indeed, many practitioners of transition metal photochemistry have for some time been interested in developing methods for converting solar radiation to chemical potential energy, for designing new photo-optical materials and for improving the efficiency of lighting with new types of OLEDs. Biomedical applications include photodynamic therapy, new photoactivated anti-cancer drugs and the photochemical delivery (photo-uncaging) of bioactive substances to physiological targets.

Our focus in this regard has been on the photo-uncaging of the small molecule bioregulators (SMBs) nitric oxide and carbon monoxide. This technique has the potential to provide unprecedented control, not only of the *location* and *timing*, but also of the *dosage* for SMB delivery. Thus, photo-uncaging has value both as an investigative tool in physiology and for its ability to affect the progression of specific disease states.

NO, formed endogenously by several isoforms of the enzyme nitric oxide synthase (NOS), has numerous roles in mammalian biology including cardiovascular regulation and immune response. Among potential therapeutic applications of controlled NO delivery are cardiovascular treatment, antibacterial control of biofilms and toxic effects on cancer cells.^78^,^92^ With regard to tumors, precise spatiotemporal control is essential, since high levels of NO can kill tissue by inducing apoptosis, but low levels may instead be proliferative.^93^ Our initial interest in NO delivery derived from its role as a radiation senistizer.^94^–^96^ Although gamma-radiation is a common treatment of malignant tumors, the hypoxic regions of tumors are more radiation-resistant than normoxic tissues.^95^ Therefore, increasing the radiation-sensitivity of a targeted site will reduce collateral damage to healthy tissue. NO is also a potent vasodilator,^91^ so release at a specific site should enhance oxygenation and, correspondingly, sensitivity of the targeted tissue.

Two themes have guided our photochemical studies of metal nitrosyls. One, described above, used flash photolysis to investigate the mechanisms of ferri- and ferro-heme reactions with NO (*e.g.*, eqn (15)).^81^ The other was to develop new photochemical precursors for NO delivery to biological targets. Early collaborative experiments with researchers at the Radiation Biology Branch of the US National Cancer Institute (NCI)^94^ involved probing γ-radiation effects on hypoxic Chinese hamster lung (V79) cells treated with the compound Na_2_[Fe_2_S_2_(NO)_4_], also know as Roussin's red salt (RRS).^97^ In the dark, RRS treatment had little effect on radiation survival rates; however, simultaneous white light irradiation led to markedly enhanced cell death attributed to radiation-sensitization by photochemically released NO (eqn (18)). Stimulated by these results, we have since explored NO photo-uncaging from other Fe/S/NO clusters,^98^ several ruthenium nitrosyls^99^ and chromium(iii) nitrite complexes.^100^ With the exception of a qualitative application of Roussin's black salt (RBS) as a light-activated NO donor for vascular relaxation,^101^ these were the first photochemical studies to focus on NO uncaging.18
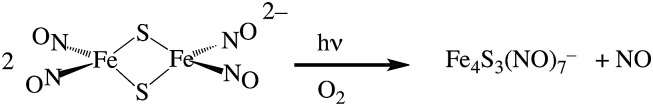



With regard to carbon monoxide, it has long been known that CO is formed endogenously during heme catabolism by the enzyme heme oxygenase.^102^ More recently, it was shown that exogenously introduced CO is cytoprotective during inflammation and promotes wound healing.^103^ Moreover, it is anti-bacterial and disrupts biofilms.^104^ In addition, CO donors called “CORMs” (CO releasing moieties) were shown in animal studies to be effective in alleviating ischemia/reperfusion (I/R) injury with various organs and tissues.^105^

Thus, for both NO and CO, there is considerable interest in developing methodologies for controlled release at targeted sites. Successful application of photo-uncaging requires elucidating both the fundamental photochemistry and photophysics of the SMB precursors and the mechanisms for transporting these species to the sites of interest. One must also recognize that mammalian tissue is a poor transmitter of shorter visible and UV wavelengths. Tissue penetration improves for longer visible wavelengths and reaches its deepest values in the near infrared (NIR) spectral region (∼700–1100 nm).^106^

**Scheme 7:**
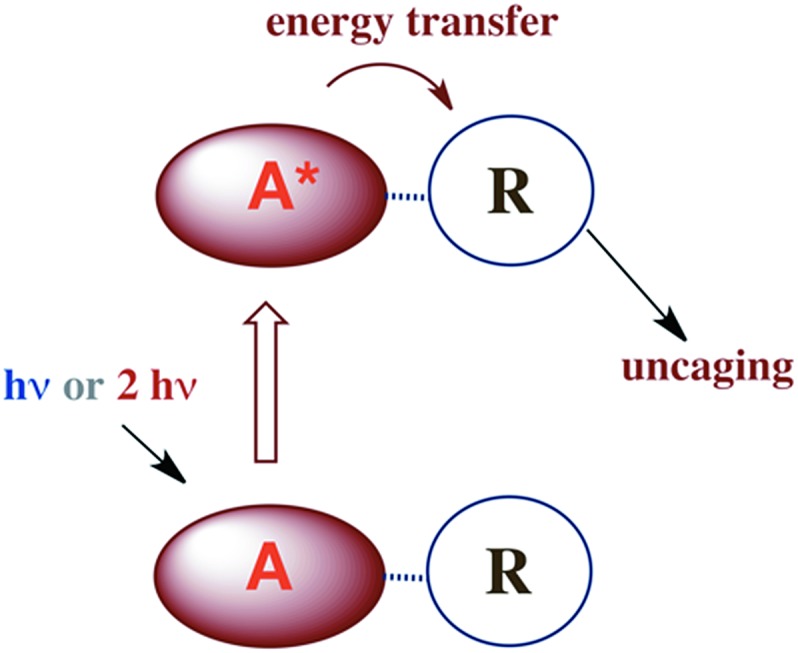
A is the antenna, R is a precursor of a SMB that is uncaged once R is photosensitized.

There are several approaches to the problem of limited tissue transmission at certain wavelengths. For example, one might design a small polymeric device attached to an implantable optical fiber.^107^ Excitation through the fiber could then provide a mechanism for delivering the caged compound upon demand. However, a less invasive approach would be to develop precursors or precursor–antenna conjugates that are responsive to single- or multi-photon excitation by tissue-transmitting light (Scheme 7).

In order to simplify terminology we will use “photoCORM”, a term we coined several years ago,^107^ as shorthand for photo-activated CO releasing moiety, and analogously, “photoNORM” for photo-activated NO releasing moiety. In designing new photoCORM and photoNORM systems, certain design guidelines are particularly attractive. Among these are (i) reasonable stability under physiological conditions (*e.g.* aerated, aqueous media at 37 °C) and (ii) the absence of undesirable toxicity of the photochemical precursor or of the residual photoproduct once the SMB is released. Also desirable are (iii) the ability to activate SMB release using longer wavelength, esp. NIR, excitation, and (iv) to track the location of these species in the organism and to determine whether the system has indeed undergone photo-activated release. In this regard, photoluminescence (PL) is a particularly sensitive imaging method. Lastly, (v) spatio-temporal control of SMB release is essential, and while this is partially achieved by using light as the trigger, developing delivery mechanisms that can target specific sites will enhance efficiency and reduce undesirable side effects.

### PhotoNORMs

Following the earlier studies referenced above, we and others developed a number of transition metal containing complexes and materials that release NO when activated by light. These studies have been summarized in various reviews,^109^ so the discussion here will be confined to systems studied in our laboratory that illustrate key aspects of the design guidelines given.

### CrONO

The chromium(iii) *O*-nitrito complex *trans*-Cr(cyclam)(ONO)_2_^+^ (“CrONO”, cyclam = 1,4,8,11-tetrazacyclo-tetradecane) meets several of the desired criteria. It is photoactive with quantum yields measured by spectral changes of 0.25–0.30 moles per einstein over the *λ*_irr_ range 365–536 nm and NO is a photoproduct.^100^,^110^ Furthermore, CrONO is stable under physiologically relevant conditions (normoxic pH 7.4 aq. buffer at 37 °C), and cell culture studies indicate no acute toxicity. Direct measurement of NO release using a Sievers Nitric Oxide Analyzer (NOA) gave *Φ*_NO_ = 0.25 when the photoreaction was carried out in the presence of added glutathione (GSH). Photolysis of low concentration CrONO solutions released sufficient NO to activate the key enzyme soluble-guanylate cyclase (s-GC) and, in separate experiments, to contract relaxed endothelium-free porcine arteries with an IC_50_ concentration ∼320 nM.110*a*
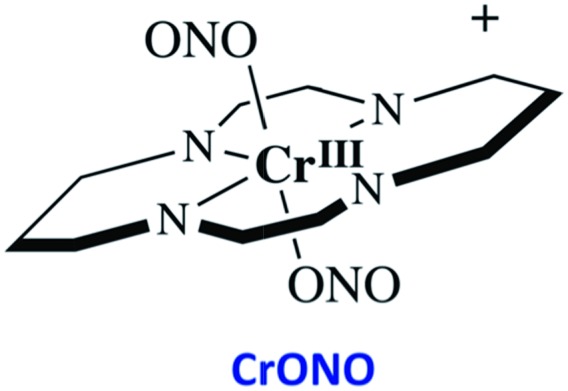


The discovery of CrONO's photochemistry was not accidental. There were previous indications that a Cr(iii) nitrito complex might undergo CrO–NO photofragmentation to give NO plus a Cr^IV^(O) species.^111^ However, photolysis of a simple complex such as Cr(NH_3_)_5_(ONO)^2+^, while showing some NO release, primarily led to ligand photolabilization.^112^ This is where knowing the photochemical literature proved valuable, since earlier studies by Kutal and Adamson had demonstrated that *trans*-Cr(cyclam)Cl_2_^+^ is relatively inert to photosubstitution, although other chlorido complexes such as Cr(NH_3_)_5_Cl^2+^ are not.^113^ These differences have been interpreted in terms of the ligand substitution from Cr(iii) MC excited states requiring substantial distortion along reaction channels involving the equatorial ligands.^6^ The relatively rigid coordination of the tetradentate cyclam ligand prevents this distortion, thereby in the case of CrONO, allows the competitive NO fragmentation pathway to dominate. DFT studies suggest that the latter occurs from the lowest energy ^2^MC state of the Cr(iii) center.110*b*

**Scheme 8:**
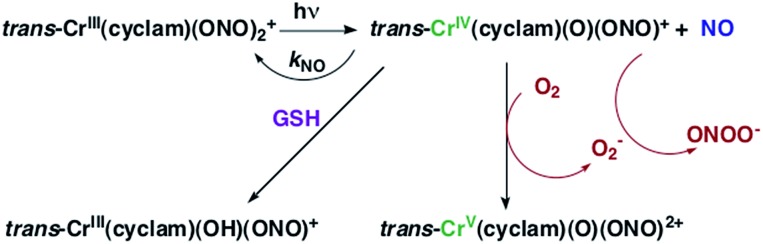
The reversible labilization of NO from CrONO. The net production of NO is effected by the trapping of the Cr(iv) intermediate by oxygen in aerobic media or by GSH in a more reducing environments.

The Cr^IV^ oxo intermediate is also quite reactive. For example, flash photolysis studies showed that it reacts with NO *via* second order kinetics (*k*_NO_ = 3.1 × 10^6^ M^–1^ s^–1^ in 298 K aq. solution) to regenerate CrONO. Permanent photochemistry is only observed when the NO is removed by entraining the solution with air or helium, or if a trapping agent intercepts the Cr^IV^(O), thereby preventing the back reaction. O_2_ serves this purpose. Thus photolysis of CrONO in aerated aq. solution leads to permanent photochemistry, presumably *via* oxidation of Cr(iv) to Cr(v). However, the net NO production is inefficient owing to scavenging by the superoxide presumably also generated in this step. On the other hand, when mM concentrations of the common cellular reductant GSH are present, reduction back to a Cr(iii) product dominates (Scheme 8).^110^

### Antennas

**Scheme 9:**
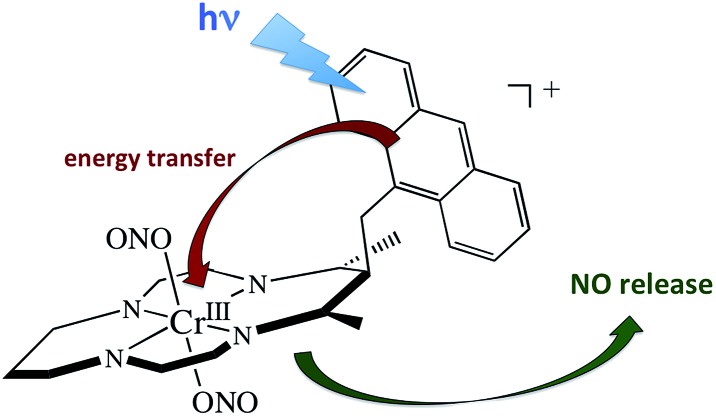
Pendant anthracene antenna collects light and photosensitize NO release from a CrONO center.

A key problem with using CrONO as a photoNORM is that its longer wavelength absorptions are ligand field bands with very low extinction coefficients (*ε*); they are Laporte-forbidden transitions for this centro-symmetric complex. Thus, the rate of NO generation is relatively slow unless the CrONO concentration and/or excitation light intensity is high. To address this issue, we decided to attach various organic chromophores to the CrONO-type platform to serve as antennas to enhance light absorption and, correspondingly, NO production (Scheme 9).^114^

**Scheme 10:**
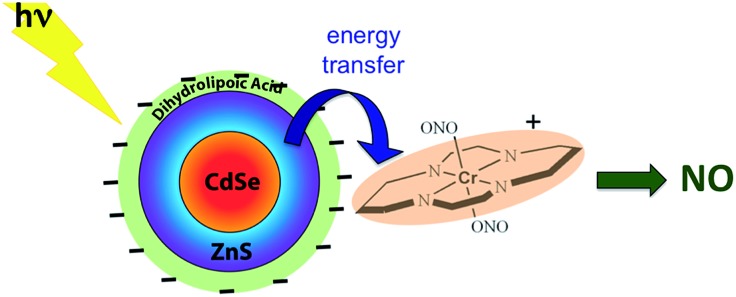
Analogous photosensitization of NO release from CrONO using a water-soluble CdSe : ZnS core : shell semiconductor quantum dot as the antenna.

While these new constructs are proof-of-principle examples of the antenna effect, they do not extend light absorption to the longer visible wavelengths needed for biological systems. For this reason we turned to semiconductor quantum dots (QDs) as photosensitizers, since QD absorption and emission energies can be tuned through the visible spectrum by varying the nanoparticle diameters.^115^ Accordingly, we prepared water-soluble CdSe : ZnS core : shell QDs by decorating the QD surface with dihydrolipoic acid (Scheme 10). Adding CrONO salts to solutions of such nanoparticles with 3.8 nm core diameters led to progressive quenching of the QD PL centered at 570 nm with increasing CrONO concentrations.^116^ More importantly, substantially more NO was photogenerated than from solutions containing only CrONO. Subsequent studies confirmed that the PL quenching and the sensitized NO production are due to Förster resonance energy transfer (FRET) from the excited QDs to CrONO.^117^ However, these electrostatic CrONO:QDs assemblies are unlikely to be stable in physiological fluids, so we are developing protocols to attach CrONO and other photoNORMS to QD surfaces *via* covalent bonds.

**Fig. 14:**
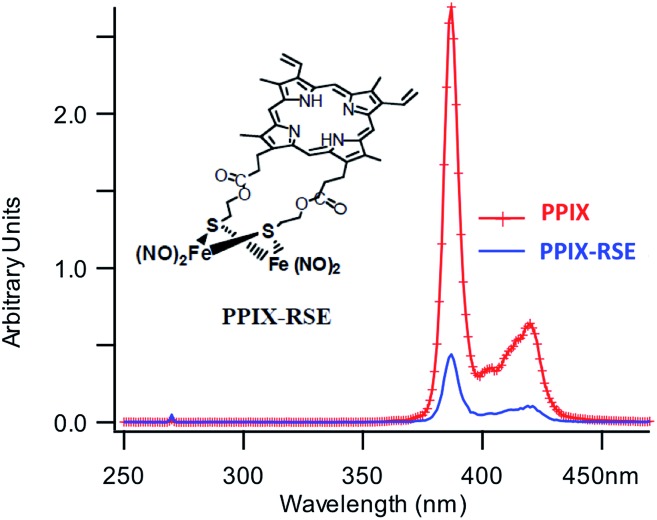
Fluorescence spectra of free PPIX and PPIX-RSE under the same conditions showing that the PPIX fluorescence is about 85% quenched by the Fe/S/NO cluster. PL lifetime measurements confirm that this quenching is due to energy transfer to the cluster (figure adapted from ref. 118).

In parallel studies, we investigated the antenna effect with Roussin's red salt esters (RSE, Fe_2_(μ-RS)_2_(NO)_4_), prepared from the RRS anion by the reaction shown in eqn (19). These RSE are more stable than is RRS and upon photolysis in aerated solution release all 4 NO (eqn (20)). Variations of this synthesis were used to build RSE's with strongly absorbing chromophores, an example being PPIX-RSE (Fig. 14).^118^ Excitation of the pendant protoporphyrin IX antenna (PPIX) photosensitizes NO release. The enhanced *rate* of NO release from PPIX-RSE *versus* that from a simpler RSE such as Fe_2_(μ-SEt)_2_(NO)_4_ (Et-RSE) is largely due to more efficient light absorption (*I*_a_) by the tethered antenna. Energy transfer from PPIX* to the cluster is also evidenced by the quenching of the characteristic PPIX PL (Fig. 14). Similar behavior was seen with Fluor-RSE, where the pendant antennas are fluorescein derivatives.^119^ Also recent study^120^ described using carbon quantum dots to photosensitize NO release from the iron sulfur nitrosyl cluster Na[Fe_4_S_3_(NO)_7_] in a nanocarrier made from carboxymethyl chitosan; however, the excitation wavelength was the near UV.19
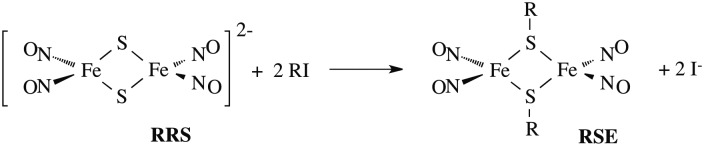

20
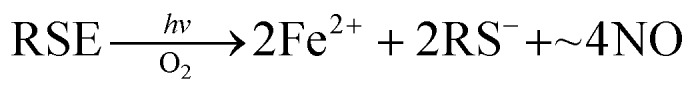



### Multi-photon excitation using NIR light

None of the photoNORMs described above displayed significant absorbance at the NIR wavelengths where tissue transmission is optimized. In principle, this limitation can be addressed by using multi-photon excitation, where the summed energy of several NIR quanta is sufficient to populate the higher energy excited states from which the desired uncaging occurs. See, *e.g.*, the report by Castellano *et al.*^121^ that NIR excitation from a fs laser triggers visible emission from Ru(ii) bipyridyl complexes. In this laboratory, we are investigating two multi-photon approaches: simultaneous two-photon excitation (TPE) and energy transfer upconversion (ETU), which functions by sequential photon absorptions. Unlike the single photon excitation (SPE) we have been discussing, the rates of TPE and ETU processes have a non-linear dependence on the incident light intensity *I*_0_. For example, the probability of TPE is proportional to *I*_0_^2^, if a single source is utilized. Thus, TPE induced photoreaction occurs mostly at the focal point of the excitation source, offering three-dimensional spatial resolution, a property widely exploited in imaging. Such resolution would provide unprecedented definition of the uncaging location.^122^ However, the high intensities needed for TPE require light sources such as pulsed lasers where very high peak powers (photons/unit time) are readily achieved. This may change with the recent description of a lower intensity mechanism for NIR to visible upconversion.^123^

The selection rules for TPE are different from those for SPE. The former is allowed only between two states that have the same parity; the latter requires a parity change.122*a* Chromophores with high TPE cross sections in the NIR include π-conjugated molecules with electron-donor and -acceptor units arranged symmetrically with respect to the center.^124^ Semi-conductor QDs are also TPE chromophores with two photon absorption cross-sections (*δ*) as large as 10^4^ GM (Goeppert-Mayer units, 1 GM = 10^–50^ cm^4^ s per photon) seen for CdSe nanoparticles.^125^

Notably, aqueous solutions of PPIX-RSE that show no absorbance at NIR wavelengths, display a weak PL (*λ*_max_ 632 nm) when subjected to 810 nm excitation with 100 fs pulses from a Ti/sapphire laser.^126^ This PL is accompanied by NO generation. These observations thus provide clear evidence that the higher energy states responsible were being accessed by multi-photon excitation, even though the *δ* value for PPIX is quite low (∼2 GM).

Fluorescein has a higher *δ* value, which was one rationale for preparing Fluor-RSE. NIR excitation of this species using an ultrafast laser gave the same PL as seen upon SPE at 436 nm, suggesting that analogous emissive excited states are formed by both methods.^127^ Like PPIX-RSE, the PL from Fluor-RSE was much less than that from the chromophores free of the Fe/S/NO cluster owing to energy transfer to the latter. The log/log plot of the NO generated *vs.* laser intensity at 800 nm light gave a slope of 1.8 ± 0.2 consistent with the (*I*_0_)^2^ dependence predicted for a photochemical process initiated by TPE.
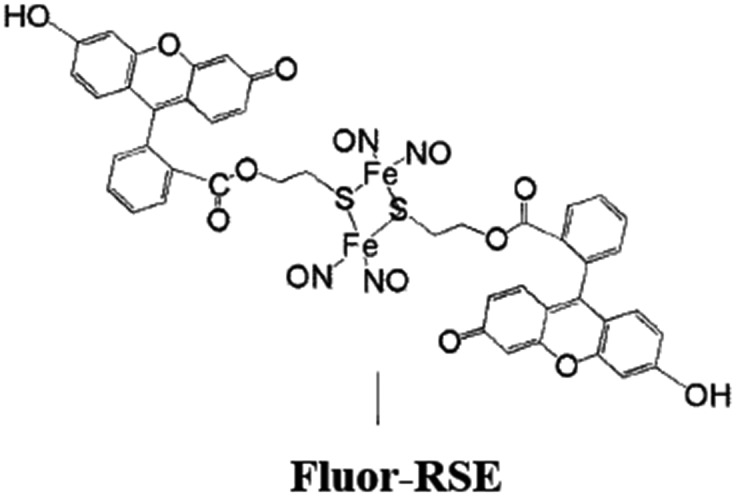


Subsequent studies have confirmed TPE with pulsed NIR lasers for other RSE derivatives with chromophores having large two photon cross-sections.^128^ Several other molecular platforms for TPE-induced NO release have also been recently described.^129^

Multi-photon NIR excitation of caged NO and other bioactive molecules can also be achieved by energy transfer upconversion. The advantage of ETU is that it involves sequential, rather than simultaneous excitation, so lower *I*_0_ values are needed. Instead of pulsed lasers, such NIR to visible/UV upconversion can be achieved with relatively inexpensive continuous wave (CW) NIR diode lasers. If the desired upconversion involves the net of two photons, the intensity dependence can vary from second order at low *I*_0_ to first order at very high *I*_0_ (where the initial excitation step becomes rate limiting). This depends upon the rates of the competing energy transfer and deactivation pathways. The typical situation is between these limits, and as a consequence, the potential for 3-D resolution using light source focusing remains.

**Fig. 15:**
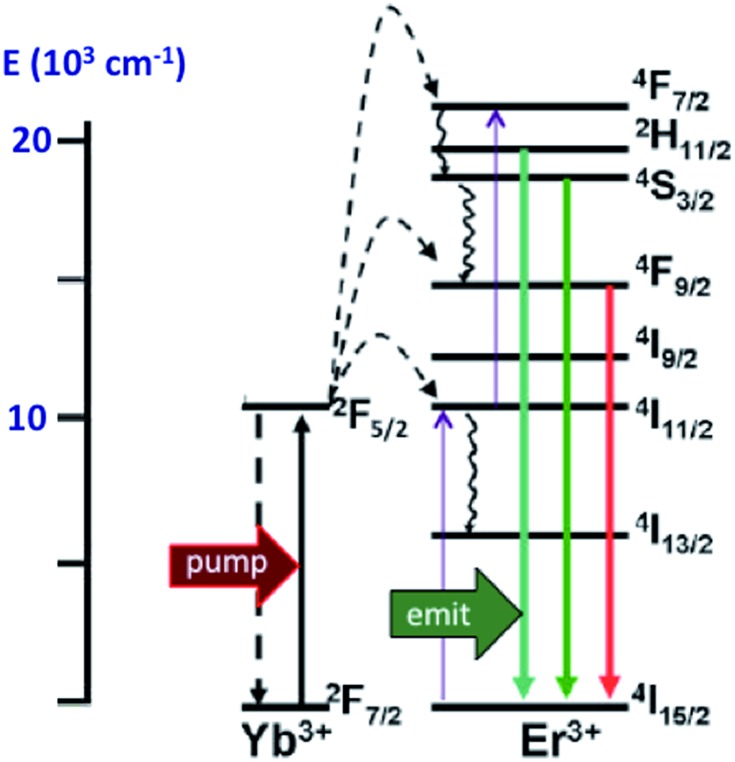
ETU mechanism for NIR of VIS upconversion using a Yb^3+^/Er^3+^ doped UCNP with a β-phase NaYF_4_ host lattice. Pumping the Yb^3+^ sensitizer with 980 nm light gives an ES that undergoes energy transfers to the Er^3+^ activator. Continued excitation of Yb^3+^ and energy transfer to Er^3+^ gives higher energy states from which several visible range emissions occur. Figure adapted from ref. 131.

In order to exploit ETU, we collaborated with Fan Zhang and Dongyuan Zhao from Fudan U. (China) and UCSB colleague Galen Stucky to test lanthanide ion-doped upconverting nanoparticles (UCNPs) as NIR antennas for NO photo-uncaging. With such UCNPs, a Ln^III^ ion such as Yb^3+^ is the sensitizer that absorbs the NIR light. This energy is transferred to an activator such as Er^3+^ as shown in Fig. 15.^130^,^131^ Further excitation of the Yb^3+^ and energy transfer to the long-lived excited state of the activator populates higher energy states from which visible/UV emission occurs. If an appropriate solid matrix is used and the core is coated with a shell of host lattice, silica or polymer, deactivation pathways are suppressed and stronger upconverted emissions result.

There was limited precedent for using such UCNPs for uncaging bioactive substances with NIR excitation. In 2010, Carling *et al.*^132^ used 980 nm photolysis of NaYF_4_ : Yb/Tm UCNPs to uncage acetate from 3′,5′-(carboxymethoxy)benzoin acetate loaded on the surface, and in 2012 Y. Yang *et al.*^133^ utilized similar UCNPs to uncage d-luciferin in C6 glioma cells and in living mice. Notably, the latter studies showed that NaYF_4_ (Yb^3+^/Er^3+^) UCNPs are *non-toxic*, their components are eventually excreted, and their location in the organism can be imaged by PL.

**Fig. 16:**
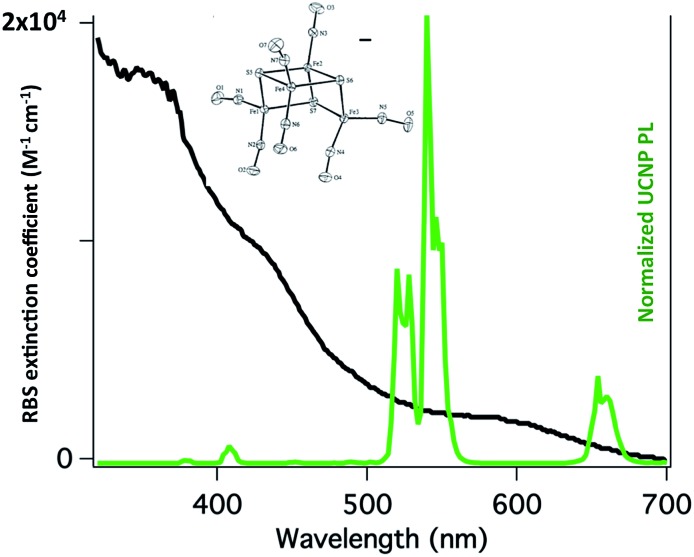
Overlap of RBS absorption spectrum and the upconverted emission spectrum from NaYF_4_ : Yb/Er (20/2%) : NaYF_4_ core : shell UCNPs excited at 980 nm.

Our first UCNP uncaging study, also reported in 2012,^134^ showed that 980 nm excitation with a CW diode laser of NaYF_4_ : Yb/Er : NaYF_4_ core : shell UCNPs triggers NO release from the anion of Roussin's black salt Na[Fe_4_S_3_(NO)_7_] (RBS). Solutions of RBS are photoactive under visible excitation (eqn (21))^98^ but show no such activity when irradiated in the NIR. This first example used UCNPs coated with a silica layer (∼10 nm) and surface modified with cationic –NH_3_^+^ groups to render the nanoparticle water-soluble and to facilitate ion pairing with the RBS anions. As shown in Fig. 16, the strong absorptions of RBS overlay the visible emissions from the NaYF_4_ : Yb/Er UCNPs, so the emitted light can be reabsorbed by RBS to facilitate the photoreaction. The NO output as measured with the NOA was linear with the irradiation time at constant power but non-linear in response to systematic increases in excitation intensity (∼*I*_0_^1.5^).21
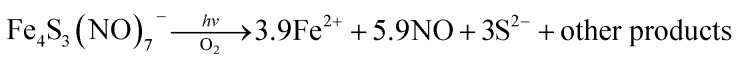



**Scheme 11:**
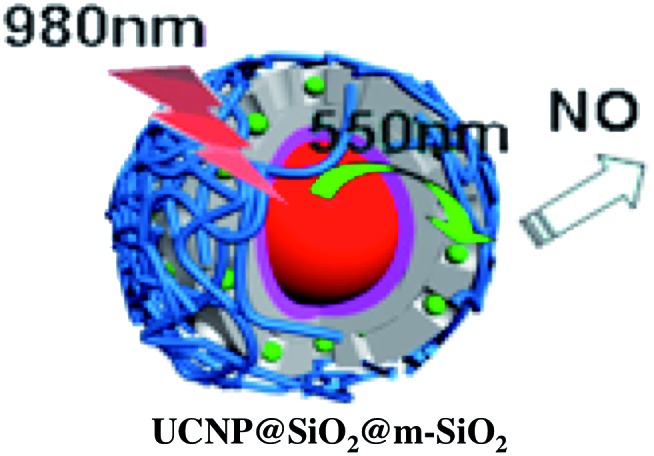
Nano-carrier with UCNP core and mesoporous silica shell impregnated with RBS (green dots) and coated with poly(allylamine). 980 nm irradiation leads to upconversion and NO release.

A more robust nanocarrier is illustrated in Scheme 11.^134^ The UCNP core was coated with silica then with a mesoporous silica shell to give a porous layer that was subsequently impregnated with RBS. Coating with poly(allylamine hydrochloride) encapsulated the photoNORM. NIR irradiation released NO, which unlike RBS can diffuse out of the nanocarrier. These proof-of-concept experiments showed that UCNPs offer the opportunity to deliver NO to specific targets using NIR light generated by a relatively inexpensive diode laser.

The mechanism by which UCNPs sensitize NIR mediated NO release from photoNORMs has not yet been well defined. Although there have been reports of Förster resonance energy transfer involving UCNPs,^135^ the photosensitization described above likely occurs by the so-called “trivial” energy transfer mechanism,^136^ namely, reabsorption of upconverted emission by the precursor owing to overlapping emission and absorption spectra as in Fig. 16. Dong *et al.*^137^ have provided convincing arguments that heating may be responsible for certain reactions sensitized by UCNPs, and this issue needs to be more thoroughly explored using temperature sensors of the type described by those workers. Defining the sensitization mechanisms that successfully utilize NIR to trigger uncaging should provide guidelines for improving the efficiency of such systems.

Although the UCNP emission/photoNORM absorption may not be the most efficient, this pathway simply requires a precursor having strong absorption bands that overlap with the UCNP emissions. This concept led our laboratory to develop biocompatible polymer-based carriers in which the UCNP and the photoNORM are simply co-dissolved. The first examples are mm-sized polymer disks consisting of polydimethylsiloxane (PDMS) containing RBS and NaYF_4_ : Yb/Er UCNPs.^138^ NO release was effected by 980 nm irradiation with a CW diode laser. Furthermore, analogous NIR excitation triggered photo-uncaging from these devices even after passing through a tissue filter. Subsequent experiments used the hydrophobic forms of Ru(salen-R)(NO)(X) and CrONO derivatives as photoNORMS in disks of various NuSIL silicone formulations.^139^ One can envision using such materials as drug delivering implants. It is also possible to miniaturize these polymer-based mini-carriers to micron and sub-micron sizes. Our on-going studies are addressing how to deliver the resulting micro- and nano-carriers with analogous formulations to physiological targets.

Another key issue in therapeutic NO delivery is detection and analysis. The Sievers NOA mentioned above measures NO that has been entrained into the gas phase from a solution or an organism. Although very accurate and sensitive, this method is transport-limited and not very useful for real time studies. There are also sensitive NO specific electrodes that are effective in solution media, although in our hands there were issues with reproducibility.98*a* Some years ago, derivatives of diaminofluorescein (DAF) were developed to provide sensitive detection of NO generation in cellular media,^140^ but it should be noted that this method actually senses the NO autoxidation product, NO_2_^–^. More direct NO detection is now available using copper(ii) complexes with chelating ligands that have pendant luminophores.^141^ The Cu(ii) largely quenches the emission from these species owing to its low energy LF transitions, but reaction with NO both modifies the ligand to enhance its PL and reduces the metal center to Cu(i), thereby deleting these low lying transitions.^142^

### PhotoCORMS

Although our investment in developing photoCORMs has been less than with NO releasing analogs, the design principles carry over as do some problems, among them the need for reliable detection methods.103d,e The standard *in vitro* test for CO is the carboxymyoglobin (Mb-CO) assay, where CO is added to a solution of reduced deoxy-Mb and formation of Mb-CO is followed *via* changes in the absorption spectrum.^143^ However, this test is not effective in aerobic media. Several fluorescent sensors have recently been reported, and this is an area of growing development.^144^,^145^ In our laboratory, we have determined photoCORM CO release by headspace analysis using both gas-phase infra-red spectroscopy and gas chromatography techniques, but found the latter to be the more reliable quantitatively.^108^ Schiller and coworkers^146^ have described headspace analysis using a standard CO gas detector. These methods were reviewed recently103d,e,^147^ and will not be discussed further here.

**Scheme 12:**
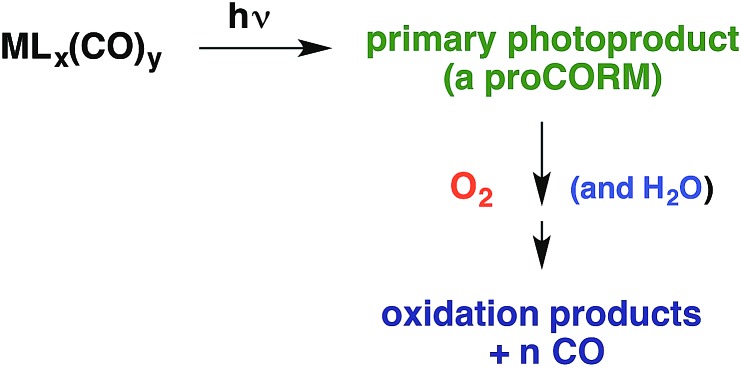
Generating a proCORM intermediate.

Our goals with photoCORMs are similar to those with the photoNORMs, namely to develop strategies to effect CO delivery to specific physiological targets. Initial studies at UCSB focused on water-soluble metal carbonyls and on improving methods for quantifying CO release.^108^,^148^ For selected group 6 carbonyls in aerated media, multiple CO's were released per complex, and the quantum yield for overall CO release (*Φ*_CO_) in some cases exceeded 1.0. The high yields of CO release can be attributed to subsequent reactions of initial photoproducts with O_2_ and/or the aqueous solvent (Scheme 12). In such instance, the primary photoproduct could be considered a “proCORM”, a molecular species that leads to slow CO release after the initial photoreaction.

**Fig. 17:**
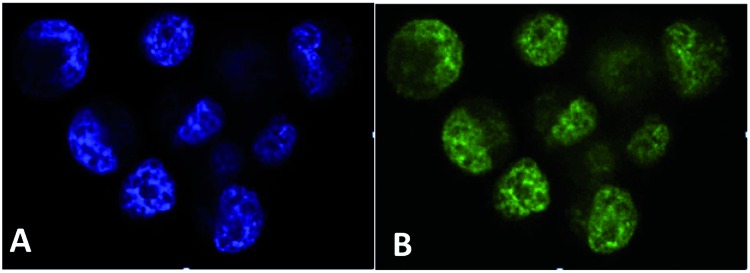
Confocal fluorescence microscopy images of PPC-1 cells incubated with Re(P(CH_2_OH)_3_)(CO)_3_(bpy)^+^ (50 μM) (A) and after photolysis at 405 nm (B) (figure adapted from ref. 149).

A very interesting photoCORM developed at UCSB is the rhenium(i) complex Re(L)(CO)_3_(bpy)^+^ (L = P(CH_2_OH)_3_).^149^ This complex ion is strongly luminescent (*τ* = 400 ns) in solution and releases one CO upon photolysis (eqn (22), *Φ*_CO_ = 0.11). The photoproduct Re(L)(CO)_2_(H_2_O)(bpy)^+^ is also luminescent. Neither Re(L)(CO)_3_(bpy)^+^ nor its photoproduct displayed any acute cellular toxicity up to 100 μM. Thus, we were able to use confocal microscopy to observe the PL of the rhenium species incorporated into cells (human prostatic carcinoma cell line PPC-1) before and after photolysis (Fig. 17). The ability to image the photoCORM in the cells and to determine whether it has undergone photoreaction is a highly desirable trait for uncaging.^150^ A major disadvantage, however, is the relatively short visible wavelengths needed to photolabilize the CO.22
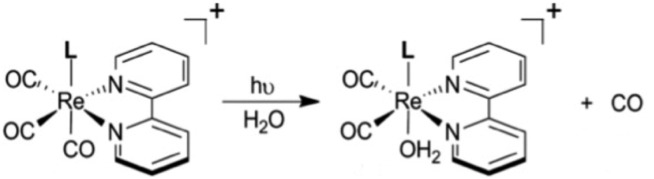



The task remains therefore to develop a mechanism to release CO with NIR light. In this context, the photophysics of metal carbonyls offers a challenge. The stronger lower energy absorptions and the PL of diimine complexes such as Re(L)(CO)_3_(bpy)^+^ can be attributed to metal-to-bipyridine charge transfer states. However, CO photolability occurs primarily from metal-centered ligand field states.^151^ Substituents can be used to tune the MLCT absorptions of the Re(i) complexes to the red, but excitation at those longer wavelength bands does not lead to significant CO release, since this does not populate the higher-energy MC states necessary for CO labilization. In fact, the story is quite analogous to the photoreactivity of the Ru(NH_3_)_5_(L)^2+^ complexes discussed above.^10^

We have addressed these issues by designing a nanocarrier that incorporates both a photoCORM and a UCNP antenna to mediate multi-photon NIR labilization of CO. The photoCORM is a Mn(i) complex similar to the Re(i) complex just described. Manganese, however, has smaller d-orbital splitting than the heavier transition element analogs. Thus, it has inherently lower energy LF states, thereby making CO photodissociation energetically more accessible.

**Scheme 13:**
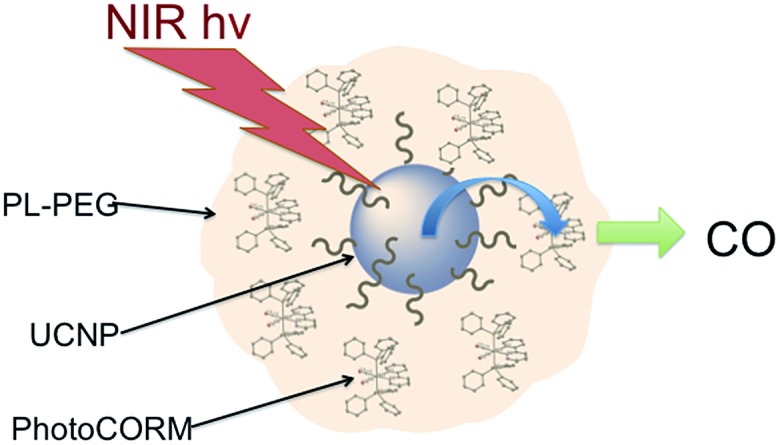
Water-soluble photoCORM nano-carriers with diameters 16–30 nm according to dynamic light scattering.

For example, the Mn(i) photoCORM *trans*-Mn(CO)_2_(PPh_3_)_2_(bpy)^+^ displays strong visible range absorptions, photolysis of which leads to facile CO release. This spectrum is well suited for matching the upconverted emissions from a NaYF_4_(Yb_20_Tm_0.2_) UCNP. In collaboration with Nanfeng Zheng of Xiamen U (China), we assembled nano-carriers consisting of a UCNP core coated with a phospholipid-functionalized with poly-(ethylene glycol) (DSPE-PEG 2000) (Scheme 13).^152^ The amphiphilic polymer confers water solubility to the nano-carrier while providing a lipid-like interior into which hydrophobic compound *trans*-[Mn(CO)_2_(PPh_3_)_2_(bpy)](CF_3_SO_3_) is readily infused. NIR excitation (980 nm) of an aq. solution containing the loaded nano-carrier leads to CO release. With these hydrophobic components, leaching is minimized, while encapsulation isolates them from the medium, thereby reducing potential toxicity and promoting higher loading. This remarkable nano-carrier brings together the UCNP NIR antenna and a hydrophobic photoCORM in a water-soluble ensemble.

We are extending these studies to examine the efficacies of such nano-carriers with other photoCORMS and photoNORMs and with UCNPs responsive to 800 nm excitation. In addition, surface modifications should be well suited for the control of targeting and other properties of these units.

### Better targeting

While photo-excitation allows one to define location and timing of SMB uncaging in tissue, a key remaining task is to improve transport of the precursor–antenna conjugates to the desired targets. Although one can envision implanting a device such as the mm-sized PDMS mini-disks described above, a more elegant strategy would be to employ biological mechanisms for this purpose. We are embarking on this quest. One approach is to use targeting peptides that, depending on the amino acid sequence, can direct water-soluble nano-carriers to specific cell types both *in vitro* and *in vivo*.^153^ For example, Tirrell *et al.*, used micelles constructed of peptide terminated polymers for the targeted delivery of chemotherapeutic agents to malignant gliomas, thereby minimizing other side effects.^154^

**Scheme 14:**
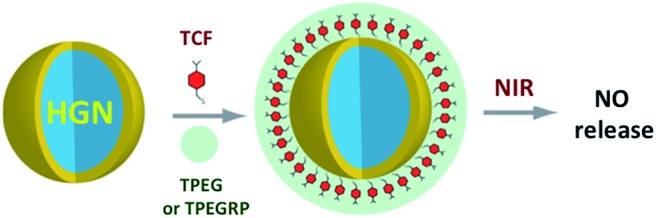
HGN surfaces are coated by co-absorption of TCF and a thiolated PEG, which enhances aqueous solubility and in the case of TPEGRP provides the cell-targeting peptide. 800 nm laser excitation of the conjugates heat the HGN surfaces and NO is released.

In collaboration with UCSB colleague Norbert Reich, we have constructed nano-carriers with hollow gold nanoparticle (HGN) cores (∼40 nm diameters).^155^ The surfaces of these HGN cores were decorated with thiocupferron (TCF), a molecule that when heated releases one equivalent of NO. Also decorating the surface was a 5 kDa thiolated polyethylene glycol (TPEGRP), which was terminated by a C-end Rule peptide, RPARPAR that targets cells with the neuropilin-1 receptor common to certain cancers. The TCF-HGN-TPEGRP conjugates prepared in this manner undergo neuropilin-1 receptor mediated endocytosis^156^ by PPC-1 and 22RV1 prostate cancer cells but not with HeLa cells, which lack that receptor. NIR irradiation with a pulsed laser at a wavelength (800 nm) resonant with the surface plasmon of the gold nanoparticles rapidly heats the nanoparticle.^157^ In solution the resulting NO release (Scheme 14) was quantified using the NOA, while intracellular NO release in 22RV1 cells was demonstrated by using the DAF-2 assay.^140^
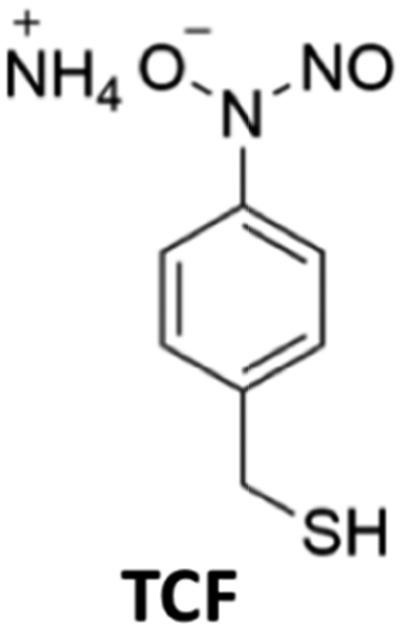


Based on this precedent, our premise is that decorating photoCORM and photoNORM nano-carriers with a targeting polypeptide will greatly enhance delivery specificity. The novelty here lies not in using these targeting polypeptides, but in combining this methodology with the uncaging of SMBs from NIR responsive conjugates with UCNP cores. The residual PL from UCNPs offers imaging opportunities as well. Such devices will provide imageable and unprecedented spatio-temporal control of SMB delivery to specific cell types.

## Concluding remarks

This article illustrates the evolution of research into the photochemistry of transition metal chemistry by tracing the author's interest and involvement in this area over four decades. Initial studies focused on correlating reaction product characterizations and quantum yields with spectroscopic properties to identify the excited states responsible for specific photochemical processes. This information was then used to tune excited state energies in order to correspondingly direct the resulting photoreactivities. Within the same time frame, related studies were directed toward elucidating specific mechanisms for excited state reactions and deactivation pathways. The availability of flash photolysis methods provided access to the dynamics and trajectories of excited state pathways as well as of subsequent reactions of transient intermediates formed. Notably, such species are often analogous to intermediates proposed for certain thermo-chemical processes, so pulsed photolysis offers an important mechanistic tool in catalysis and bioinorganic chemistry. Our personal journey in this area has turned to using photochemical methodologies for the uncaging of bioactive small molecules, with the eventual goal of establishing guidelines for therapeutic application.

Another researcher would undoubtedly provide a different perspective, given that individual's choice of other research problems and tools employed. Regardless, it is clear that the successful use of inorganic photochemistry, whether in energy science, biomedicine, or other foreseen or unforeseen application, needs to be based on a sound understanding of the fundamental principles that define the mechanisms of these systems. I am convinced that, as new tools and ideas are introduced, fascinating and unexpected developments in the photochemistry and photophysics of transition metal compounds will continue to be forthcoming.
